# Distinct Functions for Mammalian CLASP1 and -2 During Neurite and Axon Elongation

**DOI:** 10.3389/fncel.2019.00005

**Published:** 2019-01-29

**Authors:** Carmen Laura Sayas, Sreya Basu, Michael van der Reijden, Eugenio Bustos-Morán, Marcia Liz, Monica Sousa, Wilfred F. J. van IJcken, Jesus Avila, Niels Galjart

**Affiliations:** ^1^Department of Cell Biology, Erasmus Medical Center, University Medical Center Rotterdam, Rotterdam, Netherlands; ^2^Centro de Biología Molecular Severo Ochoa (CSIC-Universidad Autónoma de Madrid (UAM)), Madrid, Spain; ^3^Instituto de Tecnologías Biomédicas (ITB), Universidad de La Laguna (ULL), Tenerife, Spain; ^4^Instituto de Biologia Molecular e Celular—IBMC and Instituto de Inovação e Investigação em Saúde, University of Porto, Porto, Portugal; ^5^Center for Biomics, Erasmus Medical Center, University Medical Center Rotterdam, Rotterdam, Netherlands; ^6^Centro Investigación Biomédica en Red Enfermedades Neurodegenerativas (CIBERNED), Madrid, Spain

**Keywords:** cytoskeleton, microtubules, microtubule plus-end tracking proteins, cytoplasmic linker associated proteins (CLASPs), neuronal differentiation, axon outgrowth

## Abstract

Mammalian cytoplasmic linker associated protein 1 and -2 (CLASP1 and -2) are microtubule (MT) plus-end tracking proteins that selectively stabilize MTs at the edge of cells and that promote MT nucleation and growth at the Golgi, thereby sustaining cell polarity. *In vitro* analysis has shown that CLASPs are MT growth promoting factors. To date, a single CLASP1 isoform (called CLASP1α) has been described, whereas three CLASP2 isoforms are known (CLASP2α, -β, and -γ). Although CLASP2β/γ are enriched in neurons, suggesting isoform-specific functions, it has been proposed that during neurite outgrowth CLASP1 and -2 act in a redundant fashion by modulating MT dynamics downstream of glycogen synthase kinase 3 (GSK3). Here, we show that in differentiating N1E-115 neuroblastoma cells CLASP1 and CLASP2 differ in their accumulation at MT plus-ends and display different sensitivity to GSK3-mediated phosphorylation, and hence regulation. More specifically, western blot (WB) analysis suggests that pharmacological inhibition of GSK3 affects CLASP2 but not CLASP1 phosphorylation and fluorescence-based microscopy data show that GSK3 inhibition leads to an increase in the number of CLASP2-decorated MT ends, as well as to increased CLASP2 staining of individual MT ends, whereas a reduction in the number of CLASP1-decorated ends is observed. Thus, in N1E-115 cells CLASP2 appears to be a prominent target of GSK3 while CLASP1 is less sensitive. Surprisingly, knockdown of either CLASP causes phosphorylation of GSK3, pointing to the existence of feedback loops between CLASPs and GSK3. In addition, CLASP2 depletion also leads to the activation of protein kinase C (PKC). We found that these differences correlate with opposite functions of CLASP1 and CLASP2 during neuronal differentiation, i.e., CLASP1 stimulates neurite extension, whereas CLASP2 inhibits it. Consistent with knockdown results in N1E-115 cells, primary *Clasp2* knockout (KO) neurons exhibit early accelerated neurite and axon outgrowth, showing longer axons than control neurons. We propose a model in which neurite outgrowth is fine-tuned by differentially posttranslationally modified isoforms of CLASPs acting at distinct intracellular locations, thereby targeting MT stabilizing activities of the CLASPs and controlling feedback signaling towards upstream kinases. In summary, our findings provide new insight into the roles of neuronal CLASPs, which emerge as regulators acting in different signaling pathways and locally modulating MT behavior during neurite/axon outgrowth.

## Introduction

Neurons are highly polarized cells, with two biochemically and functionally distinct compartments emerging from the cell body: a long and thin axon that transmits signals, and multiple shorter dendrites that receive signals. This high degree of polarization is crucial for neurons to reach their proper targets and establish synaptic contacts that lead to the formation of a functional nervous system. Neuronal polarization and axon extension are largely controlled by the actin and microtubule (MT) cytoskeletons, and their associated proteins, which transduce extracellular signals into the necessary morphological changes. It is generally assumed that neurite elongation is promoted by MT stabilization (Zhou et al., 2004; Witte et al., 2008; Neukirchen and Bradke, 2011).

The dynamic behavior of the MT network is regulated by different types of MT associated proteins (MAPs), among which are the MT plus-end tracking proteins (+TIPs), a heterogeneous MAP sub-group whose members specifically associate with the ends of growing MTs (Akhmanova and Steinmetz, 2015). Most +TIPs require End Binding (EB) proteins (in particular EB1 or -3) for MT-end binding. By contrast, EB proteins themselves track MT ends autonomously (Bieling et al., 2007, 2008) and they have therefore been termed the “core” +TIPs.

Mammalian cytoplasmic linker associated protein 1 and -2 (CLASP1 and -2) are homologous +TIPs that are encoded by different genes (Akhmanova et al., 2001). Both CLASPs contain a serine/arginine-rich domain in which two short SxIP motifs are embedded, with which CLASPs interact with EB-proteins and bind MT ends (Mimori-Kiyosue et al., 2005; Honnappa et al., 2009). Interestingly, this domain is also responsible for the interaction with the scaffolding protein IQGAP1 (Watanabe et al., 2009), the cell polarity factor PAR3 (Matsui et al., 2015) and the adaptor protein Dab1 (Dillon et al., 2017). CLASPs are further characterized by a C-terminal protein-protein interaction domain, that binds partners like CLIP-115 and -170 (Akhmanova et al., 2001), LL5α and -β (Lansbergen et al., 2006), and GCC185 (Efimov et al., 2007). CLASPs also have so-called TOGL motifs, which are structurally similar to the TOG domains in XMAP215 (Al-Bassam and Chang, 2011), and which are distributed throughout the proteins. Whereas only a single CLASP1 isoform (called CLASP1α) has been described to date, three CLASP2 isoforms are known, termed CLASP2α, -β, and -γ (Akhmanova et al., 2001). The α-isoforms contain three TOGL domains of which the first is located at the N-terminus of the proteins. CLASP2β/γ lack TOGL1 and hence these isoforms only contain two TOGL domains. *In vitro* experiments suggest that CLASPs promote MT growth (Yu et al., 2016; Aher et al., 2018; Lawrence et al., 2018), and that TOGL1 might confer additional properties to CLASP-α isoforms (Yu et al., 2016).

Some of the +TIPs, including CLASPs (Akhmanova et al., 2001), Adenomatous Polyposis Coli (APC; Zhou et al., 2004), and Actin Crosslinking Family 7 (ACF7; Wu et al., 2011) can selectively stabilize MTs in specific regions of the cell upon reception of signaling cues. It is noteworthy that all these +TIPs are regulated by glycogen synthase kinase 3 (GSK3), a constitutively active kinase with a central role in neurite and axon outgrowth (Beurel et al., 2015). GSK3 inactivation results in an increased affinity of CLASP2 for MT ends (Akhmanova et al., 2001; Wittmann and Waterman-Storer, 2005) due to dephosphorylation of CLASP2 in the domain that binds EB-proteins and MTs (Kumar et al., 2009, 2012; Watanabe et al., 2009). Conversely, CLASP2 phosphorylation by GSK3 greatly impairs the ability of CLASP2 to bind MT ends. GSK3, in turn, is controlled by a number of upstream signaling molecules, for example atypical protein kinase C (aPKC), a kinase that induces neurite extension when activated (Shi et al., 2003, 2004). Most models depict a pathway in which an upstream signal leads to the inactivation of GSK3 by phosphorylation on serine 9 (for GSK3β) or 21 (for GSK3α), which in turn results in the dephosphorylation of a GSK3 target, for example a +TIP like APC (Zhou et al., 2004), allowing MT stabilization and neurite elongation.

CLASPs selectively stabilize MTs at the cell cortex in migrating fibroblasts (Akhmanova et al., 2001). They do this by forming complexes with membrane-anchored proteins such as LL5β, thereby attaching MTs to the cell cortex and promoting local MT rescue (Mimori-Kiyosue et al., 2005; Lansbergen et al., 2006). In addition, CLASPs were shown to enhance MT nucleation at the Golgi, in conjunction with GCC185 (Efimov et al., 2007). CLASP function has also been studied during neurite, axon and dendrite outgrowth; however, different results were obtained depending on the organism or neuronal cell type studied and the approach used. This has led to a somewhat confusing view in the field about the precise role of CLASPs in these processes. For example, mutations that inactivate Orbit/MAST, the single *Drosophila melanogaster* ortholog of CLASPs, caused axon guidance defects *in vivo* and led to ectopic crossing of the midline in the central nervous system (Lee et al., 2004), whereas a knockdown of Orbit/MAST in cultured *Drosophila* primary neurons revealed only a small effect on neurite outgrowth (Beaven et al., 2015). *Xenopus laevis* contains a single *CLASP* gene which encodes a protein (XCLASP1) that mostly resembles human CLASP1. The depletion of XCLASP1 was shown to hamper axon extension (Marx et al., 2013). By contrast, in mammalian neurons, knockdown of CLASP2 in cortical neurons resulted in longer axons (Hur et al., 2011), whereas in hippocampal neurons (HNs) it resulted in shorter axons (Beffert et al., 2012; Dillon et al., 2017). In addition, in dorsal root ganglia (DRG) neurons, the single knockdown of CLASP2 was less effective, but that of CLASP1 or the combined depletion of CLASP1 and -2 impeded neurite outgrowth (Hur et al., 2011).

We have studied the functions of CLASP1 and -2 in differentiating N1E-115 neuroblastoma cells, and in *Clasp2* mouse knockout (KO) neurons. We show that CLASPs have distinct localizations and roles in N1E-115 cells during neurite outgrowth, and that CLASP1 stimulates extension and CLASP2 impairs it. We also demonstrate that *Clasp2* KO HNs undergo significantly faster neurite and axon outgrowth at early stages, suggesting a role for CLASP2 as a negative regulator of neurite/axon extension. We similarly observed enhanced neurite extension in CLASP2-deficient DRG neurons, indicating that CLASP2 acts as a brake in neurite extension in different neuronal types. We attribute the opposing functions of the CLASPs to a different expression and distribution of isoforms in neurons, and a different sensitivity for GSK3. Furthermore, distinct feedback signaling towards upstream kinases, such as GSK3 and PKC, may also distinguish the CLASPs. Our data suggest that the mammalian *Clasp1* and -*2* genes have evolved to couple distinct MT behaviors to specific signaling cascades.

## Materials and Methods

### Antibodies

We used the following commercial primary antibodies (Abs): mouse anti-α-tubulin, mouse anti-β-tubulin, mouse anti-α-Tyrosinated-tubulin, mouse anti-α-Acetylated tubulin, mouse anti-β-actin, and rabbit anti-GAPDH (all from Sigma-Aldrich); mouse anti-βIII tubulin (Promega), mouse anti-α-detyrosinated (Glu)-tubulin (Abcam); mouse anti-GFP (Roche); rabbit anti-Akt-phosphoSer (Ser 473), rabbit anti-GSK3-α/β-phosphoSer (Ser 21/Ser9), rabbit anti-GSK3-α/β (Cell Signaling), rat anti-CLASP1 (#1A6) and -CLASP2 (#12H2, Absea antibodies). We also used previously described primary rabbit anti-CLASP1 [#402, for immunofluorescence (IF), and #2292 for western blot (WB)], and rabbit anti-CLASP2 (#2358, both for IF and WB; Akhmanova et al., 2001). In IF studies, anti-mouse, anti-rabbit or anti-rat secondary antibodies conjugated with either 488, 555 or 594 Alexa fluorochrome, were used (Molecular Probes). HRP-conjugated secondary antibodies used in WB assays (anti-mouse-HRP, and anti-rabbit-HRP) were purchased from Jackson ImmunoResearch.

### Cell Culture and Mice

N1E-115 mouse neuroblastoma cells (ATCC) were cultured in Dulbecco’s Modified Eagle’s Medium (DMEM) containing 10% fetal bovine serum (FBS), 2 mM of L-glutamine, 100 U/ml penicillin and 100 mg/ml streptomycin at 37°C in a 5% CO_2_ atmosphere incubator. Cell differentiation was triggered either by overnight serum starvation leading mostly to cell flattening or to neurite extension in some cells, or by culturing the cells for 6–7 days in DMEM containing 2% FBS and 1.25% dimethyl sulfoxide (DMSO), to induce a neuron-like phenotype with long and thin neurites.

Cultures of dissociated hippocampal pyramidal cells from wild-type (WT) and *Clasp2* KO mice were prepared as described (Banker and Cowan, 1977). Upon hippocampi dissection and treatment with papain and DNase (Worthington Biochemical Corporation), 10,000 cells were plated on 12 mm glass-coverslips coated with 100 μg/ml poly-L-lysine and 10 μg/ml laminin in Neurobasal medium containing 10% horse serum. Three hours after plating, medium was replaced with Neurobasal medium supplemented with 2 mM L-glutamine, 2 mM D-pyruvate, 2% B27, 100 U/ml penicillin and 100 mg/ml streptomycin. All culturing media and supplements were purchased from Gibco Invitrogen Corporation. Neuronal cultures were maintained for 1 day *in vitro* (DIV) or 2DIV in a humidified 37°C incubator with 5% CO_2_.

Primary sensory DRG neurons were isolated from 4-week-old mice as described (Fleming et al., 2009). Briefly, DRG were digested with 0.125% collagenase IV-S (Sigma-Aldrich) for 1.5 h at 37°C and centrifuged over a 15% albumin cushion for 10 min at 200 *g*. Dissociated neurons were seeded on poly-L-lysine/laminin-coated coverslips in 24-well plates at 5,000 cells per well and cultured for 12 h.

This study was carried out in accordance with European, national and local animal legislation. Protocols were approved by the Erasmus MC animal ethical committee (DEC), the IBMC Ethical Committee, and the Portuguese Veterinarian Board.

### Plasmids and Transfection

Previously described expression plasmids were used: GFP-CLASP1α, GFP-CLASP2α, and GFP-CLASP2γ (Akhmanova et al., 2001), mutant constructs GFP-CLASP2-9SA, GFP-CLASP2-8SD, and their WT control (which are slightly shorter versions of GFP-CLASP2γ; Kumar et al., 2009) and EB3-GFP (Stepanova et al., 2003). Different GFP-bearing shRNA plasmids against mouse CLASP1 or CLASP2 and a non-targeting one were purchased from Dharmacon and used in transient transfection experiments. N1E-115 cells were transfected using Lipofectamine TM 2000 (Invitrogen, Carlsbad, CA, USA), following the manufacturer’s protocol, for 48 h or 72 h, respectively.

### shRNA Constructs, Lentiviral Transduction and Generation of Stable Cell Lines

Five different mouse CLASP1 and mouse CLASP2 shRNA lentiviral constructs (Mission, Sigma-Aldrich) were used in this study. A scramble shRNA construct was used as a control. Constructs were first validated in transient transfection in N1E-115 cells and the two that were most effective in downregulating the endogenous CLASP proteins were used in each case for the lentiviral transduction. For CLASP1 we used TRCN0000253373 (5′CCGGAGATTGGAACCAGACTTATATCTCGAGATATAAGTCTGGTTCCAATCTTTTTTG3′) and TRCN0000265397 (5′CCGGTTGGGTGAACTCTAGCAATTACTCGAGTAATTGCTAGAGTTCACCCAATTTTTG3′). For CLASP2 we used TRCN0000183632 (5′ CCGGGAACTTGAAGAGACGTTAAATCTCGAGATTTAACGTCTCTTCAAGTTCTTTTTTG3′).

Subconfluent HEK-293T cells were cotransfected with each of the selected shRNA constructs along with both pCMVdR8.74 (Addgene) and pMD2G (Addgene) plasmids, using Lipofectamine TM 2000 (Invitrogen) following the manufacturer’s protocol. Viruses were collected 48 h post-transfection and used in each case to infect N1E-115 cells for 24 h. Stable cell lines were obtained upon puromycin selection (Sigma-Aldrich) for 2–3 weeks.

### Chemicals and Treatments

N1E-115 cells were treated with different chemicals to study their effects on CLASP localization. Three structurally different compounds were used to inhibit GSK3: lithium chloride (LiCl) along with myo-Inositol (as lithium also inhibits Inositol-monophosphatases; sodium chloride was used as a control), SB-216763 (Tocris), or CHIR99021 (abbreviated as CHIR, Tocris Biochemicals). The growth factors insulin (Sigma-Aldrich) and insulin-like growth factor-1 (IGF-1; Millipore) were used to study GSK3-mediated CLASP relocalization at MT plus-ends. Specific inhibitors were used to block the activity of different kinases: Wortmannin, a PI3-K inhibitor; Triciribine, an Akt inhibitor; U0126, a MEK1/2 inhibitor; and R0-318220, a PKC inhibitor. These inhibitors were all purchased from Calbiochem.

### Western Blotting and Immunoprecipitation

N1E-115 cells were washed three times with phosphate-buffered saline (PBS) and harvested in cold lysis buffer containing 1% SDS, 1 mM EDTA, 1 mM EGTA, and 25 mM Tris (pH 7.5). Cell extracts were boiled for 10 min, centrifuged for 15 min at 13,000 rpm and sonicated for 15 s. Proteins (25–50 μg) were separated by SDS-PAGE and either transferred to nitrocellulose filters or to PVDF membranes. Filters were blocked with 10% non-fat powder milk or 5% bovine serum albumin (BSA) in Tris-buffered saline (TBS)-0.1% Tween 20 (TBS-T) and incubated with primary antibodies overnight (4°C). After three washes with TBS-T, filters were incubated with the corresponding peroxidase-conjugated secondary antibody (Jackson Immunoresearch) for 1 h, and then washed again with TBS-T three times. Immunoreactivity was visualized by enhanced chemiluminescence detection (ECL, Amersham).

To immunoprecipitate proteins from transfected HEK293 cells a 15 cm plate of HEK293 cells was transfected with 10 μg of construct. After 24 h cells were harvested by centrifugation and dounced in ice-cold lysis buffer [20 mM Tris pH7.5, 150 mM NaCl, 20 mM glycerophosphate, 5 mM MgCl2, 0.1% NP-40, 5% glycerol and IX protease inhibitors (cOmplete, Roche)]. Lysate was cleared by centrifugation at 13,000 rpm for 20 min at 4°C, and the cleared extract was loaded on 100 μL of Protein-A Affi-prep beads (Bio-Rad) cross-linked to 10 μg of rabbit polyclonal GFP antibody (a kind gift of Dr. Kerstin Wendt). The extract was incubated for 2 h at 4°C, washed three times with ice-cold TBS plus 0.01% Tween 20 (TBS-T), and proteins were eluted in 100 mM Glycine, pH 2.0. Rabbit IgG (Santa Cruz, sc-2027) was used as a control.

For cross-linking antibody to beads: 100 μL of Protein-A beads were incubated with 10 μg antibody in 1 mL TBS-T for 2 h at 4°C. Beads were washed three times with 0.2 M Sodium borate (pH 9.0). Then, 1mL of 20 mM DMP (Sigma, D8388) in 0.2 M Sodium borate was added to the beads for 30 min at room temperature. Beads were washed three times in 250 mM Tris pH 8.0 and then three times with TBS-T before binding to extract.

### Immunofluorescence, Confocal Microscopy and Image Processing

N1E-115 cells or primary neurons were subject to 100% methanol-1 mM EGTA fixation for 10 min at −20°C, in some instances (depending on the primary antibody used) followed by 4% paraformaldehyde fixation for 15 min at room temperature. Cells were then permeabilized/blocked for 1 h with PBS/0.1% Triton X-100/3% (w/v) BSA. Next, cells were incubated overnight at 4° with primary antibodies (see Antibodies subheading above). After three washes with PBS, cells were incubated for 1 h with secondary antibodies (Molecular Probes). Double or triple-stained cells were analyzed with a DMRBE fluorescence microscope equipped with a Hamamatsu CCD camera or with a Zeiss LSM 510 Meta confocal microscope. ImageJ software (Schneider et al., 2012) was used to quantify CLASP comet density and length. CLASP-comet density was defined as the number of CLASP-highlighted comets counted in delimited cell areas of 100 μm^2^. Comet lengths were defined as the distances from the peak fluorescence intensity (FI) at the MT tip to the baseline lattice intensity. A number of MT-comets (specified in the Figure legends in each case) were counted in 5–10 cells per condition in each experiment. ImageJ software was also used to quantify MT stability by measuring FI of α-tyrosinated tubulin (dynamic MTs) and α-acetylated tubulin (stable MTs). For measuring neurite outgrowth in DRGs, immunocytochemistry for βIII-tubulin was performed. All neurites with a length greater than the cell body diameter were measured using ImageJ/NeuronJ (Schneider et al., 2012). Measurements were done with the observer blinded to genotypes in over 100 neurons per condition. Two independent experiments with duplicates were performed.

### Live Cell Imaging and Quantification of MT Dynamics

EB3-GFP was transfected into N1E-115 cells, and cells were filmed 24 h after transfection. Cells were either treated with insulin (5 μg/ml) for 15 min or not treated. Cells were observed at 37°C with a LSM 510 confocal laser scanning microscope (Zeiss), as described previously (Stepanova et al., 2003). The optical slice (z-dimension) was set to 1 μm in most experiments. Laser intensity and gain values were adapted to obtain optimal signal-to-noise ratios. Time-lapse series were acquired every 1 or 2 s for 60 or 30 frames, respectively. Images were recorded by using LSM 510 software. To analyze EB3-GFP comet displacements and MT growth speed, the Manual tracking plug-in of ImageJ software was used.

### RT-PCR

For RNA isolation, cells were washed with PBS and then collected in TRI Reagent (Sigma). RNA was isolated using the RNeasy Mini Kit (Qiagen). For qRT-PCR, cDNA was generated using Random Primers (Invitrogen) and Superscript IV reverse transcriptase (Thermo Fisher) according to the manufacturer’s instructions. PCR reactions were run on a C1000 Thermal Cycler (Bio-Rad) using SyBR-Green (Sigma).

### RNA-Sequencing

Purity and quality of isolated RNA was assessed by the RNA 6000 Nano assay on a 2100 Bioanalyzer (Agilent Technologies). One microgram total RNA was used as starting material for Illumina Truseq sequencing. Poly-A tail containing mRNA was purified with oligo-dT attached to magnetic beads. Subsequently, mRNA was fragmented into ~200 bp fragments followed by first-strand cDNA synthesis using reverse transcriptase and random primers. Next, second-strand synthesis was performed using DNA polymerase I and RNaseH treatment. End-repair, phosphorylation and A-tailing were carried out followed by adapter ligation, size selection on gel and PCR amplification. PCR products were purified by Qiaquick PCR purification. Samples were sequenced on HiSeq 2000 to generate 36 bp reads and a 7 bp index read. Samples were de-multiplexed and aligned to mouse build mm9 reference genome using Tophat alignment software (Trapnell et al., 2009). RNA was further analyzed using Cufflinks (Trapnell et al., 2010).

### Statistical Analysis

In each case, sets of 3–5 experiments were performed. All graphing and statistical tests were done using SPSS17 and R software. Statistical analyses were performed using parametric (Student *t*-test or ANOVA, depending on the case) and non-parametric (Mann-Whitney test) tests depending on normality of the data sets (assessed by Shapiro-Wilk). *P*-values are indicated in the graphs and *p* < 0.05 was considered to indicate a statistically significant difference (*p < 0.05; **p < 0.005; ***p < 0.0005 and ns, not significant, as stated in bar graphs). All data were expressed as the mean ± standard error of the mean (SEM), or standard deviation (SD), as stated in each case.

## Results

### Different Distribution of CLASPs in Differentiating Neuronal Cells

**Figure 1:**
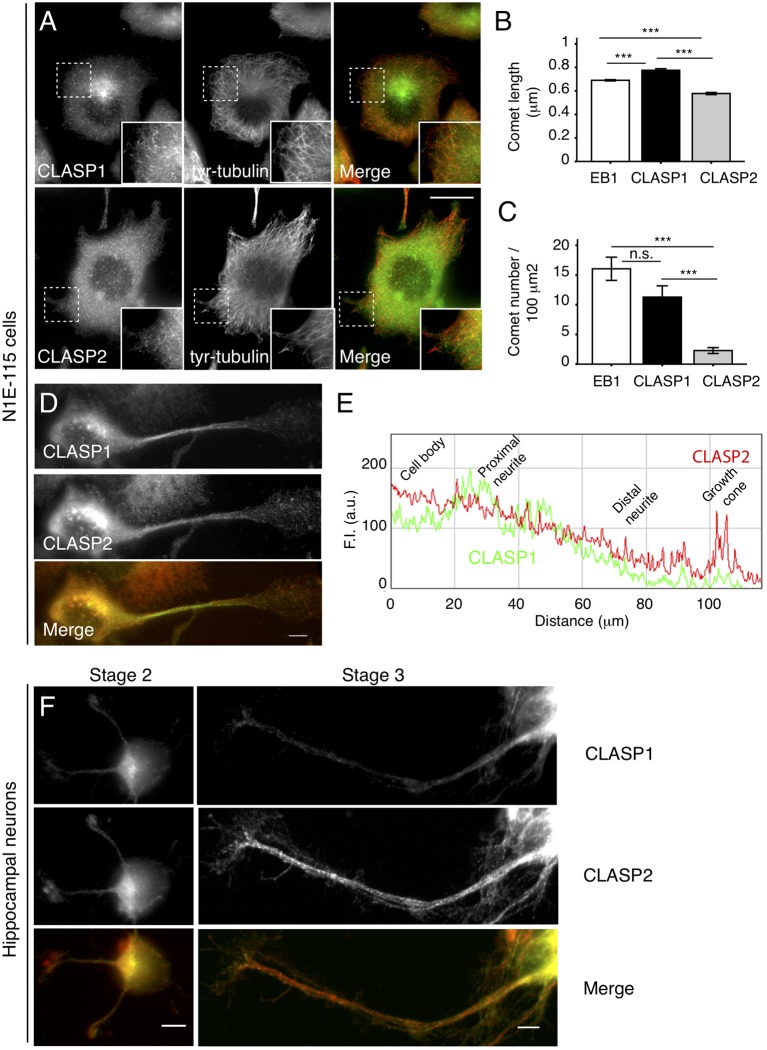
Intracellular distribution of cytoplasmic linker associated protein 1 and -2 (CLASP1 and -2) in differentiating N1E-115 cells and young primary hippocampal neurons (HNs). **(A)** CLASP1 and CLASP2 distribution in flattened N1E-115 cells. Fluorescence images of cells that were serum-deprived overnight, fixed and stained with antibodies against CLASP1 (upper left panel, green in the merged image) or CLASP2 (lower left panel, green in the merged image) and tyrosinated-tubulin (middle panel, red in the merged images). Scale bar = 20 μm. Insets show protein localization in more detail. **(B,C)** Quantification of microtubule (MT) plus-end staining (comets). Comet length **(B)** and density **(C)** of MT plus ends stained with end binding 1 (EB1), CLASP1 or CLASP2 antibodies, were measured in immunofluorescence (IF) images such as depicted in panel **(A)**. Comets were counted in 3–4 squares of 100 μm^2^ per cell. EB1: *n* = 3566 comets/24 cells; CLASP1: *n* = 699 comets/19 cells; CLASP2: *n* = 962 comets/48 cells. ANOVA tests were performed in **(B,C)**. Standard error of the mean (SEM) is depicted in each bar graph. **(D)** Localization of CLASP1 and -2 in neurite-bearing N1E-115 cells. Cells were fixed and stained with antibodies against CLASP1 (green in merge) and -2 (red in merge). In **(E)** a plot is shown with the fluorescence intensity (FI) distribution of CLASP1 (green) vs. CLASP2 (red) in process-bearing cells, from the cell body to the growth cone. **(F)** Localization of CLASP1 (green in merge) and -2 (red in merge) in mouse primary 1 days *in vitro* (DIV) HNs, at differentiation stages 2 and 3. Scale bars = 50 μm.

To address CLASP1 and CLASP2 function and behavior in neuronal cells, we used the N1E-115 mouse neuroblastoma cell line. Upon serum starvation, most of these cells flatten down; this resembles the first stages of neuronal differentiation, making N1E-115 cells a useful model in which to study the localization, function and regulation of proteins during neuronal development. Surprisingly, the localization of endogenous CLASP1 and CLASP2 at MT growing ends diverged in these early differentiating N1E-115 cells. Whereas CLASP1 was detected in a typical “comet-like” pattern on MT-plus ends throughout the cell (Figure 1A, upper panel), CLASP2 was diffuse in the cytosol and was hardly detected at MT plus-ends (Figure 1A, lower panel). We quantified CLASP staining at MT plus-ends by measuring both the length of the fluorescence intensity (FI) distribution on individual comets (expressed as comet length in μm) as well as the density of comets in cells (expressed as comet number per 100 μm^2^) using EB1 signal as a reference (see “Materials and Methods” section for an explanation on how measurements were done). We found slightly but significantly longer comets for CLASP1 (0.77 ± 0.12 μm) compared to EB1-stained comets (0.69 ± 0.005 μm), and slightly but significantly shorter ones for CLASP2 (0.58 ± 0.009 μm; Figure 1B, CLASP1 vs. EB1, *p* < 0.005; CLASP2 vs. EB1, *p* < 0.0005; CLASP1 vs. CLASP2, *p* < 0.0005). However, CLASP1 comet density was higher than that of CLASP2 (CLASP1: 13.95 ± 1.64 comets/100 μm^2^; CLASP2: 2.31 ± 0.4 comets/100 μm^2^), and almost equaled that of EB1 (19.46 ± 1.23 comets/100 μm^2^; Figure 1C, CLASP1 vs. CLASP2, *p* < 0.0005; CLASP1 vs. EB1, *p* > 0.05, and CLASP1 vs. CLASP2, *p* < 0.0005). Thus, in flat differentiating cells, CLASP1 accumulates at the distal ends of growing MTs in the cell whereas CLASP2 does not.

To examine the localization of CLASP1 and -2 during neuronal differentiation, we analyzed N1E-115 cells bearing neurites. Notably, whereas CLASP1 staining was prominent in MT plus-ends in the cell body and the proximal part of the neurite, declining progressively in intensity in the growth cone, CLASP2 localized at the ends of growing MTs along the neurite and was enriched in the growth cone (Figures 1D,E). The localization of CLASPs was also analyzed in 1DIV mouse primary HNs, a well-established system to study neuronal polarization and differentiation (Dotti et al., 1988). These neurons follow a predictable temporal sequence of morphological changes that involves initial spreading of a lamellipodium around the cell body (stage 1), which is later replaced by 3–4 minor neurites (stage 2), one of which becomes the axon (stage 3). In stage 2 neurons, CLASP2 showed a prominent localization in the distal tips of elongating neurites where CLASP1 was less abundant (Figure 1F, left panel). Stage 3 HNs showed CLASP2 enrichment in the growth cones of extending axons, while CLASP1 staining intensity was highest in the cell body and declined along the axon length (Figure 1F, right panel). Taken together these data indicate that the localization of CLASP1 and -2 proteins differs during neuronal differentiation, both in neuroblastoma cell lines and in primary neurons.

### CLASP1 and CLASP2 Are Differentially Regulated by GSK3

**Figure 2:**
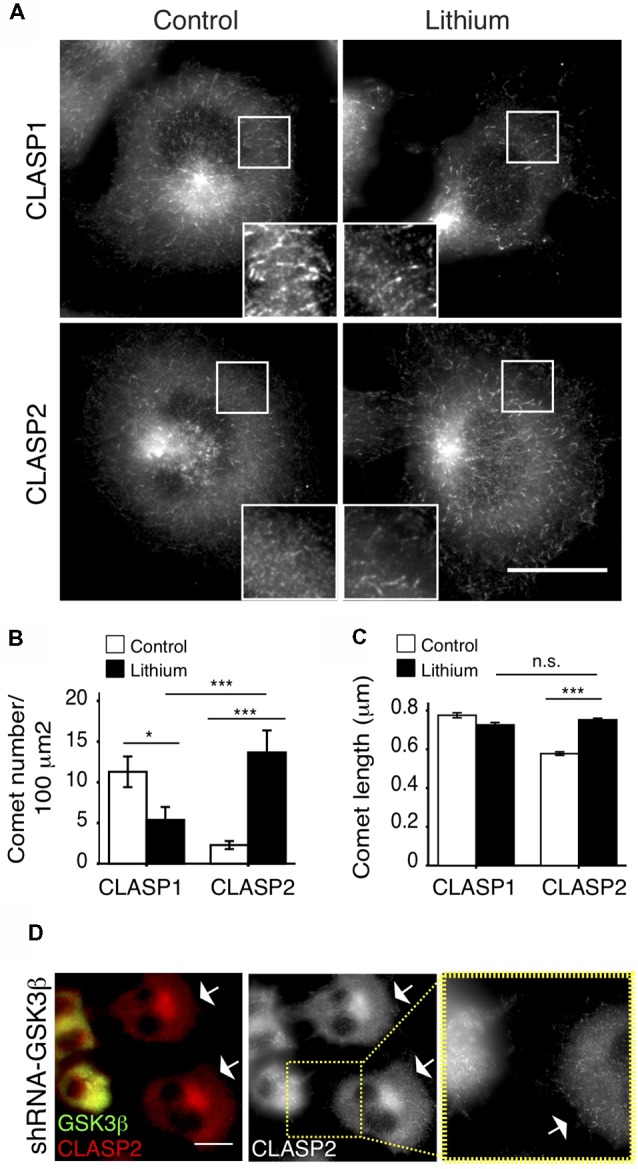
Differential regulation of CLASP1 and -2 by glycogen synthase kinase 3 (GSK3). **(A)** CLASP distribution in control N1E-115 cells and cells treated with lithium chloride (LiCl; Lithium), a well-known GSK3 inhibitor. Serum-starved N1E-115 cells were treated with Lithium (20 mM), along with myo-inositol (10 mM), for 4 h, fixed and stained with antibodies against CLASP1 (upper panels) or CLASP2 (lower panels). The control cells shown were treated with sodium chloride (20 mM) and myo-inositol (10 mM) for 4 h. Scale bar = 20 μm. **(B)** Quantification of CLASP1 and -2 comet density (number of comets/100 μm^2^) and **(C)** comet length (in μm). Comets were counted in 3–4 squares of 100 μm^2^ per cell. CLASP1: Control: 699; Lithium: 869. CLASP2: Control: 962; Lithium: 1610. Control vs. Lithium treatment was compared for each CLASP. Also, CLASP1 and CLASP2 comet density and lengths were compared after Lithium treatment in **(B,C)**. ANOVA tests were performed. SEM was depicted in each bar graph. **(D)** CLASP2 localization in N1E-115 cells upon GSK3β knockdown by shRNA, in comparison with control (non-transfected) cells. Cells were transfected with shRNA-GSK3β, fixed 72 h later and stained with antibodies against GSK3 (green in merge) and CLASP2 (red in merge). White arrows point to non-transfected cells. Scale bar = 20 μm. Inset shows detailed CLASP2 pattern in control and GSK3 knockdown cell.

CLASP2 binding to MTs was shown to be regulated by GSK3 (Akhmanova et al., 2001; Wittmann and Waterman-Storer, 2005; Kumar et al., 2009; Watanabe et al., 2009). Because of the similarity of the two proteins in the region that is phosphorylated by GSK3 (Kumar et al., 2012), CLASP1 was assumed to react similarly to this kinase during neurite outgrowth (Hur et al., 2011). However, when we treated N1E-115 cells with LiCl (Figure 2A) or SB-216763 (Supplementary Figure S1A), two different inhibitors of GSK3, the responses of CLASPs in their localization clearly differed. CLASP2, which was mostly diffuse in the cytosol before treatment, became clearly detectable at MT plus-ends upon GSK3 inhibition, while CLASP1 remained accumulated at MT ends (Figure 2A and Supplementary Figure S1A). Quantification revealed a dramatic increase in the number of CLASP2-stained comets per 100 μm^2^ upon LiCl treatment (Control: 2.5 ± 0.8 comets/100 μm^2^; LiCl: 13.7 ± 2.5 comets/100 μm^2^, *p* < 0.0005), yet a decrease in the number of CLASP1-stained comets (Control: 11.25 ± 2 comets/100 μm^2^; LiCl: 5.02 ± 1.8 comets/100 μm^2^, *p* < 0.05; Figure 2B). Moreover, CLASP2 comets were slightly but significantly longer upon GSK3 inhibition (Control = 0.578 ± 0.09 μm; LiCl = 0.752 ± 0.08 μm, *p* < 0.0005), while CLASP1 comet length did not change (Control = 0.776 ± 0.01 μm; LiCl = 0.726 ± 0.01 μm, *p* > 0.05; Figure 2C). Of note, after LiCl treatment, the density of CLASP2 comets was higher than that of CLASP1 (*p* < 0.05; Figure 2B), whereas comet length was equal for CLASP1 and CLASP2 (*p* > 0.05; Figure 2C). Consistent with these results, the knockdown of GSK3β led to an increased accumulation of CLASP2 at MT ends (Figure 2D).

The differential GSK3-mediated phosphorylation of CLASP1 and CLASP2 was further analyzed by western blot. For this experiment N1E-115 cells were first serum-starved overnight and subsequently either treated for 3 h with the GSK3 inhibitor CHIR99021, or not treated (control cells). Cell lysates were then examined for the presence of posttranslationally modified forms of CLASP1 and -2 using specific antibodies. In control cells we detected two CLASP2 bands, which we assume to represent phosphorylated and non-phosphorylated forms of CLASP2α (Supplementary Figure S1B, left panel). In cells treated with CHIR99021 only a lower band was detected, i.e., non-phosphorylated CLASP2. We only detected one CLASP1 protein, irrespective of whether cells were treated with CHIR99021 or not (Supplementary Figure S1B, right panel). These data are consistent with the immunofluorescent staining patterns of CLASP1 and -2, and suggest that in N1E-115 cells CLASP2 is a GSK3 target, whereas CLASP1 is not.

Ensembl database searches revealed *Clasp1* mRNAs which either contain or lack an internal small exon encoding 36 aminoacids. Interestingly, this exon covers the second SxIP domain of CLASPs with surrounding conserved multiple GSK3 phosphorylation consensus sites described in CLASP2 (Kumar et al., 2009). Using RT-PCR on mRNA derived from N1E-115 cells we examined possible alternative splicing of this exon in the *Clasp1* gene. Results indeed suggest that in N1E-115 cells the SxIP-containing *Clasp1* exon is mostly missing (Supplementary Figure S1C). Lack of this small region in CLASP1 might explain why this protein is not sensitive to GSK3 in N1E-115 cells.

We next investigated the localization of GFP-tagged CLASP1 and -2 isoforms. We examined N1E-115 cells expressing GFP-CLASPs at low levels because at high expression levels the GFP-CLASPs started to bind along the MT lattice and even bundled them (data not shown). Importantly, the patterns of the different GFP-tagged isoforms resembled those of the endogenous proteins, i.e., in control cells GFP-CLASP1α localized to MT plus ends, whereas GFP-CLASP2α and -γ were largely diffuse in the cytosol (Supplementary Figure S2A Control-left panels, and Supplementary Figures S2B,C). Upon LiCl treatment, the localization of GFP-CLASP1α did not change much compared to the control situation; by contrast GFP-tagged CLASP2α and 2γ changed localization, from diffuse cytosolic to MT plus-ends (Supplementary Figure S2A, Lithium-medium panels and Supplementary Figures S2B,C). Thus, the distribution of GFP-tagged CLASPs mimics that of the endogenous proteins, both in the control situation and upon GSK3 inhibition. Taken together, our results suggest that in neuroblastoma cells CLASPs are differentially regulated by GSK3.

### Regulation of CLASP Distribution by Insulin and IGF-1

**Figure 3:**
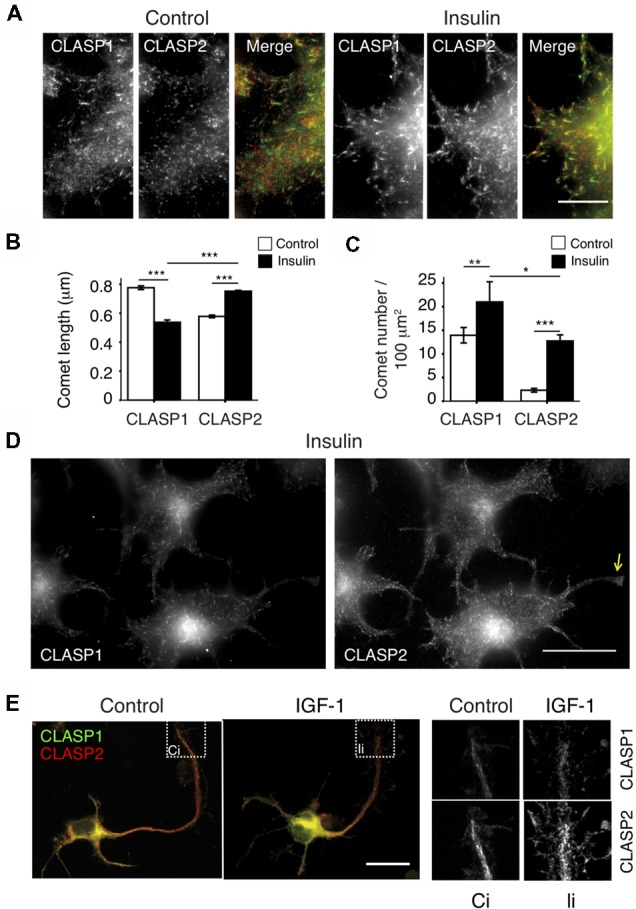
Differential regulation of CLASP1 and -2 by Insulin and insulin-like growth factor-1 (IGF-1). **(A)** Insulin regulates CLASP1 and -2 distribution differently. Serum-starved N1E-115 cells were either treated with vehicle (Control) or insulin (2 μg/ml for 10 min), and subsequently fixed and stained with antibodies against CLASP1 (green in merge) and CLASP2 (red in merge). Double IF confocal images of endogenous CLASP1 and CLASP2 at edges of control and insulin-treated cells are shown. Scale bar = 20 μm. **(B,C)** Quantification of MT plus-ends. Average CLASP comet lengths **(B)** or MT density (number of MT plus-ends stained by CLASPs per 100 μm^2^, **C**) before and after insulin addition are depicted ± SEM. Comets were compared in Control cells vs. Insulin-treated cells for each CLASP. CLASP1 and CLASP2 comet lengths and density were also compared post-treatment. ANOVA tests were performed. **(D)** CLASP2, but not CLASP1, localizes prominently in neurite tips and growth cones in insulin-treated N1E-115 cells, as shown in serum starved cells, fixed and stained with anti-CLASP1 and CLASP2. Yellow arrow points to a neurite tip. Scale bar = 20 μm. **(E)** IGF-1 induces CLASP2 relocalization to enlarged growth cones in 1DIV mouse HNs. Neurons were fixed and stained with anti-CLASP1 (green in merge) and anti-CLASP2 (red in merge). Insets show details of CLASPs localization in growth cones in control (Ci) and IGF-1-treated (Ii) neurons. Scale bar = 20 μm.

CLASP2 plays a role in the stabilization of a subset of MTs oriented towards the leading edge of migrating fibroblasts (Akhmanova et al., 2001; Drabek et al., 2006), and accumulates at MT ends upon GSK3 inhibition in N1E-115 cells. These cells have been shown to form extensive lamellipodia and membrane ruffles in response to insulin (van Rossum et al., 2003), a well-known physiological inducer of GSK3 inhibition (Welsh and Proud, 1993). Therefore, insulin seemed a good candidate regulator of CLASP function by GSK3 in our cell model. After confirming the lamellipodia-forming effect of short insulin treatments (2 μg/ml for 10 min, data not shown), the distribution of each CLASP was analyzed and compared in control vs. insulin-treated cells. Insulin addition induced CLASP2 binding to MT plus-ends throughout the cytoplasm (Figures 3A,D), with a prominent localization of long CLASP2-positive comet dashes on cortical regions proximal to cell edges. Interestingly, CLASP2 comets were longer after insulin addition (Control: 0.58 ± 0.009 μm; Insulin: 0.75 ± 0.006 μm, *p* < 0.0005), whereas CLASP1 comets became shorter (Control: 0.77 ± 0.01 μm; Insulin: 0.54 ± 0.02 μm, *p* < 0.0005; Figure 3B). CLASP2 comet density was significantly and dramatically enhanced in response to insulin (Control: 2.31 ± 0.4 comets/100 μm^2^; insulin: 12.74 ± 1.3 comets/100 μm^2^, *p* < 0.0005), while the density of CLASP1-positive dashes was also augmented, but to a lesser extent (Control: 13.95 ± 1.64 comets/100 μm^2^; insulin: 21 ± 4.2 comets/100 μm^2^, *p* < 0.005; Figure 3C). Comparison between CLASP1 vs. CLASP2 comets after treatment revealed that CLASP2 comets were slightly but significantly longer than CLASP1 comets (*p* < 0.0005) whereas the density of CLASP1 comets was higher than that of CLASP2 (*p* < 0.05). Insulin exerted similar effects on GFP-tagged CLASP1 and CLASP2, leading to a clear relocalization of GFP-CLASP2 isoforms α and γ at cortical MT plus-ends (Supplementary Figure S2A, insulin right panel, and Supplementary Figures S2B,C). In addition, whereas CLASP2 was clearly detected in nascent growth cones and neurite tips in insulin-treated N1E-115 cells, CLASP1 accumulated less prominently in these regions (Figure 3D).

IGF-1 evokes similar signaling cascades as insulin (Laurino et al., 2005) and has similar effects on CLASP2 localization in N1E-115 cells (data not shown). Since IGF-1 has been shown to stimulate neuronal polarity, axon extension and membrane expansion in primary neurons (Feldman et al., 1997; Pfenninger et al., 2003), we checked the effect of adding IGF-1 to 1DIV mouse HNs on CLASP localization. As described (Pfenninger et al., 2003), IGF-1 induced an expansion of the growth cone area, and we observed a concomitant redistribution of CLASP2 (Figure 3E). Therefore, CLASP2 distribution in growth cones can be regulated by extracellular signals involved in neuronal polarity and neurite extension. In summary, our data show that insulin provokes an overall accumulation of CLASP2 at MT-plus ends, with more and longer comets. This effect is different for CLASP1, with increased number but shorter CLASP1-stained comets, pointing to insulin as a differential regulator of CLASP function in neuronal cells. IGF-1 resembles insulin in its actions on CLASP localization in 1DIV hippocampal primary neurons.

Acetylation is a posttranslational modification (PTM) on tubulin that is often used as indicator of MT stability (Cambray-Deakin and Burgoyne, 1987). In control N1E-115 cells, only a few acetylated MTs were present, localized in the perinuclear region together with CLASP2 (Supplementary Figure S3A, upper panel). After insulin addition, cells showed a dense array of acetylated MTs oriented toward the lamellipodia cell edge, which correlated with CLASP2 comets (Supplementary Figure S3A, lower panel), similar to what occurs in polarized migrating cells (Akhmanova et al., 2001). Insulin also induced a net increase in acetylated tubulin in these cells (Supplementary Figure S3B). We counted the total number of growing MTs per μm^2^ before and after insulin addition, using an antibody against EB1, and found that insulin did not significantly alter the amount of growing MTs; however, the length of EB1 comets was reduced after insulin treatment (data not shown). In confocal time-lapse experiments we confirmed that EB1-GFP comets were shortened in response to insulin (data not shown), and that MT growth speed, as measured by tracking EB1-GFP comets, was reduced (*p* < 0.05; Supplementary Figure S3C). Taken together these results indicate that in serum-starved N1E-115 cells insulin induces the stabilization of a subset of MTs that are oriented towards the lamellipodial cell edge and that become acetylated. The accumulation of CLASP2 at the ends of growing MTs in insulin-treated cells correlates with a reduced MT growth rate, suggesting an important role for CLASP2 in the dampening of MT polymerization speed. These data are consistent with *in vitro* properties reported for CLASPs, which have been shown to reduce MT growth rate and cause persistent MT growth (Yu et al., 2016; Aher et al., 2018; Lawrence et al., 2018).

### Feedback Signaling by the CLASPs

**Figure 4:**
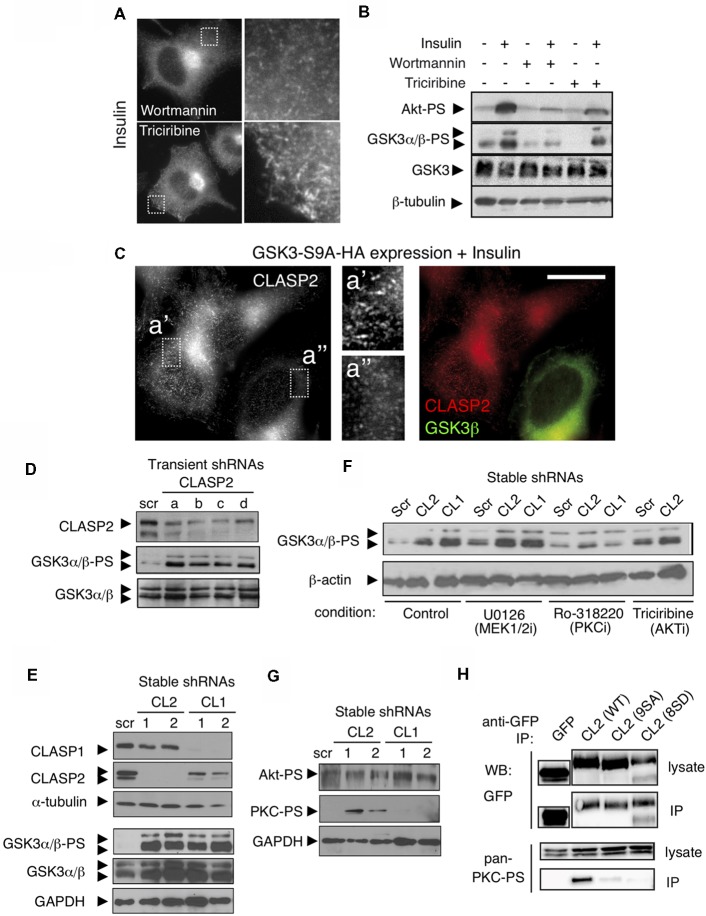
Knockdown of the CLASPs reveals a role in feedback loop signaling. **(A)** Inhibition of PI3K but not of Akt, blocks Insulin-induced binding of CLASP2 to MT plus-ends. CLASP localization was assessed by IF in serum starved insulin-treated (2 μg/ml for 10 min) N1E-115 cells, pretreated with Wortmannin (PI3K inhibitor, 100 nM, 30 min) or Triciribine (Akt inhibitor, 10 μM, 30 min). **(B)** Insulin induces an increase in serine phosphorylation of GSK3α/β, downstream of PI3K but not of Akt, in N1E-115 cells. Western blots (WBs) show Insulin-mediated signaling in serum starved N1E-115 cells, pretreated with PI3K or Akt inhibitors. We used antibodies against Akt-PS (Ser473), GSK3α/β-PS (Ser9/Ser21), total GSK3 and β-tubulin. **(C)** Active GSK3β abolishes CLASP2 relocalization to MT plus-ends induced by Insulin. N1E-115 cells were transfected with constitutively active GSK3β (GSK3β-S9A-HA), treated with Insulin as above, fixed 48 h post-transfection, and double stained with anti-HA (green in merge, to stain active GSK3β) and anti-CLASP2 (red in merge). **(D)** Transient knockdown of CLASP2 increases GSK3 serine phosphorylation. N1E-115 cells were transfected with the indicated shRNA constructs. After 72 h, cells were lysed and the levels of the indicated proteins examined by WB. **(E)** Stable depletion of CLASP1 and -2 enhances GSK3 serine-phosphorylation. N1E-115 cells were transfected with the indicated shRNA constructs and selected with puromycin. Resistant clones were picked and examined for CLASP1 or -2 knockdown. In two of each, we next examined the levels of phosphorylated and total GSK3 by WB. **(F)** Inhibition of protein kinase C (PKC) alleviates CLASP-depletion-mediated GSK3 phosphorylation. Stable N1E-115 clones expressing the indicated shRNAs were treated with inhibitors (i) against MEK1/2 (U0126, 1 μM, 30 min), PKC (Ro318220, 0.5 μM, 30 min), or Akt (Triciribine, 10 μM, 30 min). Cells were subsequently lysed and the levels of the indicated proteins examined by WB. **(G)** Depletion of CLASP2—but not of CLASP1—leads to increased phosphorylation of atypical PKC (aPKC) but not of Akt. N1E-115 clones stably-depleted in CLASP1 or CLASP2 were lysed and the levels of the indicated phospho-proteins (Akt-PS and PKC-PS) were examined by WB **(H)** Interaction of CLASP2 with phosphorylated aPKC. N1E-115 cells were transiently transfected with wild type (WT) GFP-CLASP2 [CL2 (WT)], GFP-CLASP2-9SA, or with GFP-CLASP2-8SD. After 1 day, cells were lysed and the GFP-tagged forms of CLASP2 were pulled-down with anti-GFP antibodies. Lysates and co-IPs were examined by WB using antibodies against the indicated proteins.

Since the effect of insulin on CLASP2 binding to MT plus ends was more pronounced than on CLASP1 in N1E-115 cells, we focused on CLASP2 for our subsequent signaling analysis. N1E-115 cells were pretreated with inhibitors of either PI3-K (Wortmannin or LY-294002) or Akt (Triciribine) in order to assess their role on insulin-mediated CLASP2 relocalization to MT plus-ends. Both Wortmannin and LY-294002 abolished insulin-induced CLASP2 accumulation at MT plus tips (Figure 4A, upper panel, and data not shown), concomitant with an inhibition of lamellipodia formation as previously reported (van Weering et al., 1998). However, Akt inhibition did not abolish but enhanced the insulin-induced increase in CLASP2 comet density (Figure 4A, lower panel, compare with Figures 3A,D). WB analysis revealed the correct action of the respective inhibitors on PI3K and Akt in N1E-115 cells (Figure 4B). Of note, while Wortmannin blocked GSK3 serine phosphorylation and its concomitant inhibition, Triciribine did not abolish the increase in GSK3-PS levels, suggesting first that Akt is not involved in insulin-mediated GSK3 inhibition in N1E-115 cells and second that GSK3 inhibition is crucial for CLASP2 relocalization after insulin treatment. To confirm the latter point, a constitutively active mutant of GSK3β (Ser9 replaced by Ala, and therefore non-phosphorylatable) was expressed in N1E-115 cells and the effect of insulin on CLASP2 was tested. In insulin-treated and GSK3β-S9A-HA transfected cells, CLASP2 was mainly diffuse in the cytoplasm as compared with the surrounding non-transfected cells in which CLASP2 accumulated at MT growing ends (Figure 4C). These results indicate that insulin-induced GSK3 phosphorylation and inhibition leads to CLASP2 relocalization downstream of PI3-K, but these processes are not mediated by Akt.

The observations that insulin treatment gives rise to lamellipodia and stable MTs, that insulin-mediated CLASP2 relocalization to MT plus-ends involves inactivation of GSK3, and that CLASP2 is a direct substrate of GSK3 (Kumar et al., 2009) are all consistent with CLASP2 acting downstream of GSK3. However, the possibility that CLASP2 might crosstalk with GSK3 has never been tested. For that purpose, we examined whether knockdown of CLASP2 caused GSK3 serine phosphorylation. We transiently depleted CLASP2 in N1E-115 cells using four independent shRNAs. Strikingly, in each case we found a significant increase in the phosphorylation state of GSK3α/β (Figure 4D). We extended these studies to N1E-115 cells that were stably depleted of either CLASP1 or CLASP2. Depletion of either CLASP induced an increase in the level of phosphorylated GSK3α/β (Figure 4E). We next tested the effect of inhibitors of three important GSK3 regulators (i.e., MEK1/2, PKC, and AKT). Inhibition of MEK1/2 or AKT did not affect the increased phosphorylation of GSK3 after CLASP depletion (Figure 4F). By contrast, inhibition of PKC significantly impaired this effect both after CLASP1 and CLASP2 knockdown (Figure 4F). Finally, we observed that knockdown of CLASP2—but not of CLASP1—caused the phosphorylation, and thus activation, of PKC (Figure 4G).

CLASP2 has been shown to interact with the cell polarity factor PAR3; this interaction is required for association with, and phosphorylation by, aPKC (Matsui et al., 2015). It is noteworthy that the interaction with PAR3 is in the GSK3-sensitive S/R-rich domain of CLASP2 that is also responsible for the interaction with EB-proteins. Mutant forms of CLASP2 have been made, in which all GSK3 phosphorylation sites were either mutated to alanine (9SA, phosphorylation-resistant) or aspartic acid (8SD, phosphorylation-mimic); the association of these mutants with MT ends was shown to be enhanced or reduced, respectively, as compared to WT protein (Kumar et al., 2009). We tested whether the interaction between CLASP2 and aPKC was influenced by these mutations by performing immunoprecipitations in cells expressing WT GFP-CLASP2, GFP-CLASP2-9SA, or -8SD, and examining co-precipitation of active (i.e., phosphorylated) aPKC. Interestingly, we only detected an interaction between WT GFP-CLASP2 and phospho-aPKC, but not with either of the mutant GFP-CLASP2 proteins (Figure 4H). These data suggest that modification of the GSK3 sites in the S/R domain of CLASP2 abrogates the interaction with active aPKC.

Our combined data suggest that the shRNA-mediated depletion of either CLASP1 or CLASP2 causes serine phosphorylation of GSK3; in addition, CLASP2 depletion also results in the phosphorylation and activation of PKC, another kinase with which CLASP2 appears to interact. Thus, CLASP1 and CLASP2 are not only substrates of different kinases (e.g., GSK3 and PKC) but they also act upstream of these kinases in distinct feedback loops that regulate kinase activities.

### Distinct Roles for CLASP1 and -2 in Neurite Outgrowth in N1E-115 Cells

**Figure 5:**
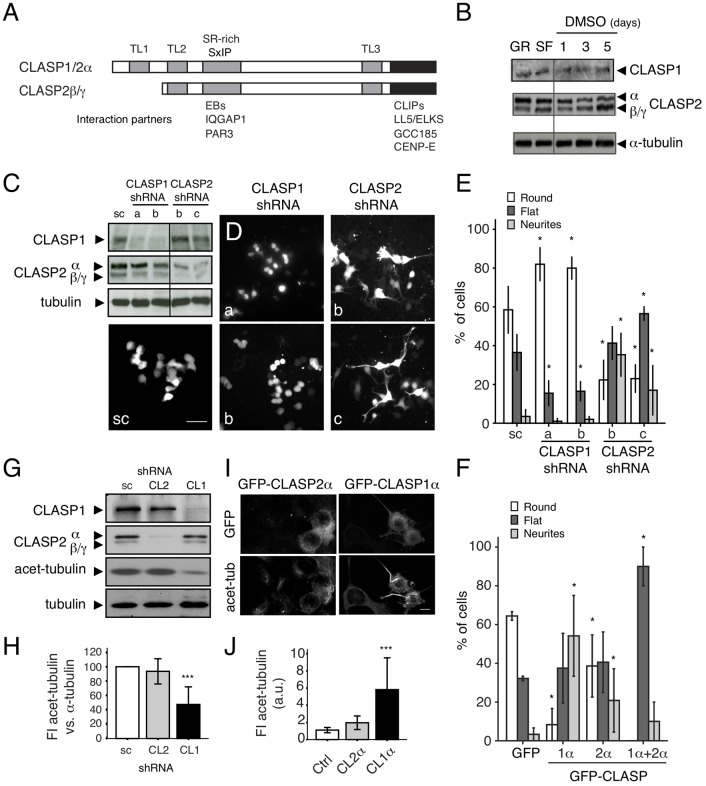
Opposing roles of CLASP1 and CLASP2 in N1E-115 cells. **(A)** Schematic representation of CLASP domains and isoforms. The position of the TOGL (TL) and serine/arginine-rich (SR-rich) motifs is indicated by gray bars. Interaction partners and the regions in CLASPs with which they interact, are shown underneath. **(B)** CLASP1 and -2 expression (WB) in N1E-115 cells before and after differentiation, by overnight serum deprivation (SF for Serum Free) or with dimethyl sulfoxide (DMSO) for 1, 3, and 5 days. **(C,D)** Transient knockdown of CLASP1 and -2 in N1E-115 cells. WBs of lysates **(C)** and IF staining in N1E-115 cells **(D)** transfected with a scrambled shRNA construct or with different shRNA constructs targeting CLASP1 or CLASP2 (a and b for CLASP1 and b and c for CLASP2). Cells were either lysed for WB or fixed for IF, 72 h post-transfection. Cells were not serum-starved but were maintained in 10% fetal bovine serum (FBS)-containing medium. In **(C)** we used anti-α-tubulin as a loading control. In **(D)**, Scale bar = 50 μm. shRNA constructs bear GFP as a reporter. **(D,E)** In the IF experiments, GFP-expressing CLASP1-depleted cells present mostly round morphology, whereas CLASP2-depleted cells show a differentiated morphology (flat or with neurites). This was quantified in E. Number of cells with each different morphology (round, flat or neurite-bearing cells) was compared in cells transfected with either CLASP1-or CLASP2-shRNAs vs. the scramble shRNAs (scramble/control). Student *T*-tests were performed. The effects had different extents depending on the shRNA used. **(F)** Overexpression of GFP-CLASP1α induced dramatic neurite outgrowth and GFP-CLASP2α only moderate neurite extension in N1E-115 cells. Transfected N1E-115 cells were counted for the presence of round, flat and neurite-bearing cells. Comparisons for statistical analyses were done between control cells (transfected with GFP) and cells transfected with either CLASP1α, CLASP2α, or CLASP1α+CLASP2α. Student *T*-tests were performed. Standard deviation (SD) is depicted in the bar graphs in **(E,F)**. **(G–J)** MT stability in CLASP knockdowns. **(G)** Lysates of N1E-115 cells stably depleted of either CLASP1 or CLASP2 were examined by WB, using antibodies against CLASP1, -2 and acetylated-tubulin (stable MTs). Total α-tubulin and GAPDH were used as loading controls. **(H)** Densitometric quantification of acetylated-tubulin vs. α-tubulin in representative WBs of control stable cells (scr) or cells deficient in CLASP1 or -2. **(I)** IF confocal images show N1E-115 cells transfected with GFP-CLASP1α or GFP-CLASP2α and stained for acetylated tubulin. **(J)** Quantification of FI in arbitrary units (a.u.) of acetylated tubulin in control (non-transfected) cells and cells ectopically expressing GFP-CLASP2α or GFP-CLASP1α from representative fluorescent images. SD is depicted in bar graphs of **(H,J)**.

Our findings show that, in differentiating neuronal cells, CLASP1 and CLASP2 display different MT binding behavior and subcellular localization, are differently regulated by GSK3, and activate distinct signaling pathways and feedback loops, when knocked-down. All these data suggest that CLASP1 and -2 play distinct roles during neurite outgrowth and are non-redundant. To address this issue, we first examined the expression of CLASP isoforms (Figure 5A) in N1E-115 cells. We found that under basal (i.e., non-differentiating) conditions, N1E-115 cells expressed CLASP1α, CLASP2α, and CLASP2β/γ (Figure 5B, note that CLASP2β/γ cannot be distinguished by WB). An RT-PCR-based analysis suggested approximately equal levels of the *Clasp* mRNAs (Supplementary Figure S4). Neuronal differentiation induction [either by overnight serum starvation (SF) or by DMSO-containing medium for 1, 3, and 5 days] led to an increase in the level of CLASP2β/γ compared to CLASP2α (Figure 5B), which is consistent with whole brain extract results (Drabek et al., 2012), and indicates a specific role for the shorter isoforms of CLASP2 during neurite extension in N1E-115 cells.

We next transiently depleted CLASP1 and -2 using two different GFP-bearing shRNA constructs for each protein (Figure 5C and Supplementary Figure S5A). Interestingly, in cells cultured under basal conditions, the knockdown of CLASP1 resulted in an increase in the amount of round (undifferentiated) cells and a decrease in the percentage of flat cells and cells with neurites (in differentiation), whereas depletion of CLASP2 caused a reduction in cell rounding with a concomitant cell flattening, and increase in the number of cells with neurites (Figures 5D,E, and Supplementary Figures S5A,B). Overexpression of GFP-tagged CLASP1 in non-differentiating cells led to an increase in the number of cells with neurites, whereas overexpression of CLASP2 only caused a moderate increase (Figure 5F). Co-expression of CLASP1 and 2 induced a huge rise in the percentage of flat cells and disappearance of round cells (Figure 5F).

Since an increase in MT stabilization (and hence acetylated tubulin levels) has been associated with neurite/axon outgrowth during neuronal differentiation (Witte et al., 2008) and CLASPs have been shown to locally regulate MT stability (Drabek et al., 2006), we checked whether the differences exerted by CLASP1 and -2 on neurite outgrowth correlated with a distinct action of CLASPs on tubulin acetylation. Both transient (Supplementary Figure S5B) and stable knockdown of CLASP1 caused a decrease in the level of acetylated α-tubulin (Figures 5G,H and Supplementary Figures S5C,D), whereas overexpression increased the amount of this marker of stable MTs (Figures 5I,J). By contrast, knockdown of CLASP2 did not change the level of acetylated tubulin (Figures 5G,H), and CLASP2 overexpression only mildly increased it (Figures 5I,J). Taken together these data indicate that mammalian CLASPs have opposing roles during neurite outgrowth of N1E-115 cells, with CLASP1 stimulating extension and CLASP2 hampering it. Thus, even though mammalian CLASPs are highly similar proteins their function in neurite elongation and effects on MTs appear to differ.

### Neurite and Axon Extension Are Accelerated in *Clasp2* Knockout Neurons

**Figure 6:**
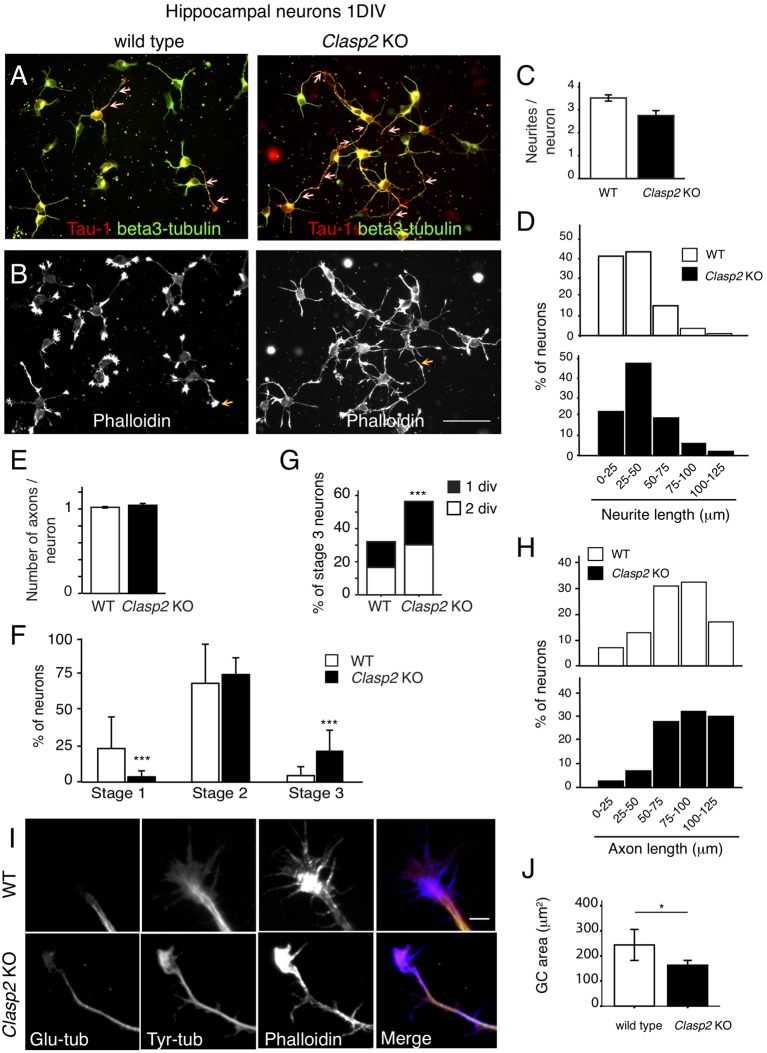
*Clasp2* knockout (KO) neurons develop longer neurites and show accelerated axon outgrowth. **(A,B)** WT and *Clasp2* KO HNs were cultured for 1 day *in vitro* (1DIV), fixed and stained with antibodies against Tau1 (an axonal marker, red in merge), βIII-tubulin (a neuronal marker, green in merge; **A**) and phalloidin (F-actin; **B**). **(C)** Quantification of the mean number of primary neurites/neuron in WT and *Clasp2* KO neurons. No differences were found. Student *T*-test was performed. SD is depicted. **(D)** Histograms showing the distribution of primary neurite length in WT and *Clasp2* KO neurons. The latter present the same number of primary neurites, but these are longer. **(E)** Quantification of the number of axons/neuron reveals no differences between WT and *Clasp2* KO neurons. **(F–H)** *Clasp2* KO neurons develop premature and longer axons. In **(F)** the percentage of neurons in stage 1, 2 or 3 (at 1DIV) was quantified and represented in histograms. In **(G)**, the percentage of stage 3 neurons was quantified in 1 and 2DIV WT vs. *Clasp2* KO neurons. In **(H)** the histograms represent the distribution of axon lengths of WT and *Clasp2* KO neurons. **(I,J)** Growth cone morphology in the absence of CLASP2. The actin and MT cytoskeleton were visualized in 2DIV WT and *Clasp2* KO neurons using anti-α-detyrosinated-tubulin (Glu-tub, green in merge) and anti-α-tyrosinated-tubulin (Tyr-tub, red in merge) antibodies, in combination with phalloidin (F-actin, blue in merge). Scale bar = 10 μm. In **(J)** a quantification of growth cone area is shown using phalloidin staining. WT neurons have wider growth cones with more polymerized actin than *Clasp2* KO neurons. Student *T*-test was performed. SD is depicted in bar graph.

To analyze the role of CLASP2 in primary neurons we cultured embryonic HNs (Supplementary Figure S6), derived from WT and *Clasp2* KO mice (Drabek et al., 2012). HNs were first analyzed after 1DIV, using antibodies against βIII tubulin and Tau1 to monitor neurite extension and axon formation, respectively (Figure 6A), and phalloidin to examine the actin network (Figure 6B). This analysis revealed that neurite extension was enhanced and axon formation was accelerated in *Clasp2* KO neurons (Figures 6A,D,F). In addition, actin protrusions were diminished in *Clasp2* KO neurons (Figure 6). Quantifications revealed that the average number of neurites present in CLASP2-deficient neurons remained unchanged but their length was enhanced (Figures 6C,D), as compared with WT neurons. The lack of CLASP2 did not seem to affect neuronal polarity, as *Clasp2* KO neurons had one axon per neuron, like control cells (Figure 6E). However, we found a significant increase in the number of CLASP2-deficient neurons that had reached stage 3 of differentiation after 1DIV (Figures 6F,G) and 2 DIV (Figure 6F), and more neurons with longer axons (Figure 6H). Hence, 1DIV and 2DIV *Clasp2* KO neurons show an accelerated initiation of axon extension and present longer neurites and axons.

Since we observed an enriched localization of CLASP2 in WT neurite tips, and axonal growth cones (see Figure 1F), we analyzed MT and actin cytoskeletons in distal axons and growth cones of 2 DIV hippocampal WT and KO neurons. In WT neurons, MT bundles were located in the center of the growth cone, with some individual MTs entering the peripheral domain (Figure 6I). In *Clasp2* KO neurons, both dynamic and stable MTs (stained with antibodies recognizing tyrosinated or detyrosinated tubulin, respectively) entered more extensively into the actin-rich domain of the growth cone, with more overlap between tyrosinated and detyrosinated MTs, and actin filaments (Figure 6I). Significant effects were found in growth cones of *Clasp2* KO neurons, i.e., growth cones were smaller, and not only less well spread and simpler and with fewer filopodia than WT growth cones, but they also contained reduced amounts of polymerized actin (Figures 6I,J). These results indicate that both actin and MT dynamics are locally altered in growth cones of *Clasp2* KO neurons.

We next examined DRG neurons after 4 DIV and found that CLASP2 depletion did not affect neurite formation, as the percentage of cells bearing neurites was similar to WT DRG neurons (data not shown) and total neurite length remained unchanged (Supplementary Figure S7A). However, *Clasp2* KO DRG neurons displayed an increase, both of the length of the longest neurite and in the mean neurite length (Supplementary Figures S7B,C), and a decreased segment number (Supplementary Figure S7D), suggesting that in normal DRG neurons CLASP2 restricts axonal elongation in favor of axonal branching. Overall, our data suggest that deficiency in CLASP2 leads to longer neurites, and axons tipped by smaller growth cones, in correlation with alterations in MT and actin growth cone networks. These results point to CLASP2 as a brake for neurite and axon extension in different types of neurons.

We have shown previously that CLASP2 is most abundantly expressed in the brain (Akhmanova et al., 2001), and that CLASP2β/γ are the major isoforms present in brain protein extracts (Drabek et al., 2012). Next generation sequencing of RNA (RNA-Seq) from 3DIV HNs suggested increased *Clasp2*β/γ levels compared to *Clasp2α*-mRNA (Supplementary Figures S8A,B). Moreover, RNA-Seq showed that both in hippocampal and in DRG neurons *Clasp2* mRNA levels are higher than *Clasp1* (Supplementary Figure S8C). Given the relative enrichment of CLASP2 in growth cones, as shown by immunofluorescent staining experiments (Figures 1F, 3E), these data suggest that the amounts of CLASP2β/γ in this neuronal compartment might exceed the level of CLASP1α.

## Discussion

Mammalian CLASPs are homologous proteins that are often similarly localized and have overlapping functions in non-neuronal cells (e.g., Mimori-Kiyosue et al., 2005). It has been proposed that CLASP1 and CLASP2 are also redundant in neurons (Hur et al., 2011). Here, we reveal distinct functions for the CLASPs in N1E-115 neuroblastoma cells with CLASP1 stimulating neurite outgrowth and CLASP2 acting as a break. Results in primary *Clasp2* hippocampal and DRG KO neurons support a role for CLASP2 as an antagonist of neurite and axon outgrowth.

CLASP2 has been shown to be phosphorylated by GSK3 on different serine residues in the S/R-rich region surrounding two SxIP motifs (Kumar et al., 2009, 2012; Watanabe et al., 2009). Since CLASP1 is quite similar to CLASP2 it is generally thought that CLASP1 and -2 are regulated in the same manner by GSK3. However, our data suggest that CLASP2 is a major target of GSK3β in N1E-115 cells and neurons, while CLASP1 is not. For example, in serum-starved N1E-115 cells CLASP2 was hardly localized at MT plus ends indicating high GSK3 activity (which is confirmed by low GSK3-serine phosphorylation in these cells), and globally relocalized to MT-plus ends throughout the cell after GSK3 inhibition or knockdown. By contrast, GSK3 inhibition induced a modest reduction in CLASP1 binding to MT-growing ends. Moreover, WB analyses also indicated that in neuroblastoma cells CLASP2 is phosphorylated by GSK3 but CLASP1 is not. Thus, in N1E-115 cells CLASP1 and -2 display different sensitivity towards GSK3. One reason for this different behavior could be that in N1E-115 cells the majority of *Clasp1* mRNAs lack an internal small exon encoding the second SxIP domain of CLASP1 with many of the GSK3 phosphorylation consensus sites.

We also observed CLASP2 relocalization to MT plus-ends upon insulin or IGF-1 treatment in neuroblastoma cells or neurons, respectively. Both growth factors induce GSK3 inhibition corroborating that CLASP2 binding to MTs in neuronal cells is modulated by different signaling pathways that converge at the GSK3 level. Remarkably, in L6 myotubes, CLASP2 has been shown to be phosphorylated and to participate in the activation of MT-based glucose transport in response to insulin (Langlais et al., 2012), supporting a role for CLASP2 in insulin signaling in different cell types. Moreover, while CLASP2 behavior downstream of insulin mimics that after GSK3 inhibition in our cell system, CLASP1 does not respond equally to both stimuli. This suggests that CLASP1 localization might be modulated by different kinases and/or phosphatases regulated by insulin.

In our experiments GFP-tagged CLASP1 and -2 localized and behaved like endogenous proteins. This reinforces our antibody-based results, and also suggests that GSK3 phosphorylation interferes with GFP-tagged CLASP2—but not GFP-CLASP1—interaction with MT plus-ends. Mild overexpression of GFP-CLASP2 in differentiating cells will therefore initially yield phosphorylated protein, and this form of GFP-CLASP2 is not able to stabilize MTs. By contrast, mild overexpression of GFP-CLASP1 yields molecules that are not phosphorylated and that can increase the amount of stable (acetylated) MTs. These observations explain results shown in Figures 5I,J. It should be noted that GFP-CLASP1 does contain the small exon encoding the second SxIP motif, and hence resembles CLASP2 in this area of the protein. Thus, besides alternative splicing, other mechanisms can also underlie differential sensitivity of CLASP1 and -2 to GSK3. As GSK3 requires a “priming” kinase to function optimally, one possibility is that priming sites in CLASP1 and -2 differ.

Another surprising finding of our work is that knockdown of either CLASP1 or CLASP2 leads to the serine phosphorylation of GSK3. We have previously shown that the RNAi-mediated depletion of 4.1R, a CLASP2 interaction partner, also results in enhanced GSK3 phosphorylation (Ruiz-Saenz et al., 2013). CLASPs seem to act as regulators of GSK3 by modulating the activity of upstream factors, such as serine/threonine kinases or phosphatases. In line with this, we show that inhibition of PKC -but not of ERK or Akt-blocks GSK3 phosphorylation induced by CLASP depletion. This suggests that CLASP downregulation induces GSK3 phosphorylation by PKC. Interestingly, we observe that knockdown of CLASP2—but not of CLASP1- results in the phosphorylation and activation of aPKC. CLASP2 has been shown to be phosphorylated by—and interact with—active forms of PKC (Lanza et al., 2010). Moreover, a crosstalk between CLASP2 and the polarity complex PAR3/PAR6/aPKC has been reported: CLASP2 interacts directly with PAR3 and this interaction is required for CLASP2 phosphorylation by aPKC (Matsui et al., 2015). We here show a GSK3-dependent CLASP2/aPKC interaction, since only WT CLASP2 binds active aPKC while CLASP2 mutants in the GSK3 phosphorylation sites do not, neither the phosphomimetic nor the non-phosphorylatable forms. A possible explanation for this is that the non-phosphorylatable form of CLASP2 interacts more strongly with GSK3, as the kinase makes a vain attempt to phosphorylate CLASP2; GSK3 binding in turn inhibits PAR3 interaction and hence the association between CLASP2 and aPKC. Of note, GSK3 has been reported to interact with, and be regulated by, Par6/aPKC, leading to the recruitment of APC, another +TIP, to MT plus-ends during centrosome reorientation (Etienne-Manneville and Hall, 2003). Thus, a complex interplay between CLASP2, GSK3 and aPKC seems to exist, in which CLASP2 acts both as substrate and upstream regulator of both kinases. The fact that CLASPs appear to be part of a network involved in feedback loop signaling to GSK3 and other upstream kinases such as PKC indicates that CLASPs play a complex regulatory role in signal transduction. Part of CLASP function may be to facilitate the formation of signaling complexes. These findings have consequences for the interpretation of results of other studies in which CLASPs were depleted using RNAi. It will be interesting to examine whether other +TIPs (e.g., APC and ACF7) control upstream kinases and if so, which cascades are involved.

We describe here that CLASP proteins display opposite functions during neuronal development, with CLASP1 promoting neurite extension and CLASP2 acting as a negative regulator of neuritogenesis/axonogenesis. A model incorporating our results, including feedback loop signaling mediated by CLASPs, is schematized in Supplementary Figure S9. In neurite-bearing N1E-115 cells as well as in primary HNs, CLASP2 accumulates at MT plus-ends along the neurite/axon shaft and in the growth cone, whereas CLASP1 is enriched at MT growing ends present in soma and proximal region of neurites/axon. The levels of CLASP2β/γ increase during neurite extension whereas CLASP2α levels diminish; since CLASP2β appears to be membrane-bound (Akhmanova et al., 2001), we propose that in growth cones CLASP1α and CLASP2γ are the main CLASP proteins present, with CLASP2 being more prominent than CLASP1 (Supplementary Figure S9). This is interesting in view of a recent report, which suggests that the N-terminal TOGL1 domain of CLASP2α exerts an auto-regulatory effect, releasing the inhibitory action of the C-terminal region on the TOGL2 domain, which is proposed to be responsible for the MT catastrophe suppressor function of CLASP2 (Aher et al., 2018). Thus, CLASP2γ, which lacks the TOGL1 domain, would only be able to suppress catastrophes when the C-terminal domain interacts with other proteins, for example when CLASP2γ is bound to the cell cortex *via* interaction partners like LL5β (Lansbergen et al., 2006). During neurite/axon extension, different growth factors engage to growth cone receptors triggering diverse signaling pathways that converge at GSK3 phosphorylation and inactivation in the growth cone. Switching GSK3 from an active to an inactive form affects CLASP2 but not CLASP1 function, and it may even increase local CLASP2 concentration, as the protein attaches both to MT ends and the cell cortex and becomes less diffusive. Competition of CLASP2γ with CLASP1α at MT ends may lead to local MT stabilization in growth cone regions where GSK3 is inactive, whereas persistent MT growth, mediated by CLASP1α, is observed in growth cone regions where GSK3 is active, and in proximal neurite/axon shafts where it is enriched (Supplementary Figure S9). An extra layer of complexity arises when taking into account that CLASP2 is a direct GSK3 target in neuronal cells and a binding partner and modulator of aPKC, and that both CLASPs are upstream regulators of GSK3. Since several MT-interacting proteins, both classical MAPs and +TIPs, are direct GSK3 targets, the indirect output of CLASP actions on MT dynamics becomes even more complex. Thus, the final outcome of CLASP actions in neuronal development will most likely depend on a finely tuned balance between their expression levels, subcellular localization, as well as their involvement in signaling feedback loops.

Our *Clasp2* knockdown data in N1E-115 cells as well as the *Clasp2* KO results in primary HNs are consistent with a previous study that showed increased axon outgrowth in cortical neurons in which CLASP2 was depleted by shRNA and RNAi-mediated approaches (Hur et al., 2011). However, these authors did not observe any effect on axon extension upon CLASP1 downregulation. Moreover, they showed that in DRG neurons the single knockdown of CLASP2 was less effective, but that of CLASP1 or the combined depletion of CLASP1 and -2 impeded neurite outgrowth (Hur et al., 2011), contrary to the increase in neurite length that we show in DRG neurons obtained from our *Clasp2* KO mice. Our data do not concur with other reports in which knockdown of CLASP2 was shown to impede, rather than enhance, neurite extension in HNs (Beffert et al., 2012; Dillon et al., 2017). One reason for the different outcomes in RNAi/shRNA-mediated knockdown studies involving the CLASPs may be a varying interference with feedback loop signaling networks, which may produce different outcomes in terms of the cytoskeletal organization involved in neurite/axon extension. For example, we show here that CLASP2 depletion affects GSK3 and aPKC. The latter is known to promote axon elongation (Shi et al., 2003, 2004). Inhibition of GSK3 has also been reported to induce axon outgrowth (Kim et al., 2006); this was shown to partly be due to the enhanced binding of some of its substrates, such as APC or CRMP2, to MTs, thereby controlling MT dynamics and stability in the axon and growth cone (Zhou et al., 2004; Yoshimura et al., 2005).

*Clasp2* KO neurons present longer axons tipped by smaller growth cones, with altered MT and actin cytoskeletons. This suggests that CLASP2 deficiency might lead to local changes in MT and actin dynamics in the growth cone. Absence of CLASP2 would allow CLASP1 to occupy MT ends, and promote sustained MT growth in peripheral regions of the growth cone. This would explain our observation that in *Clasp2* KO neurons dynamic (tyrosinated) MTs appear to penetrate the peripheral regions of the growth cone more often, probably contributing to the enhanced extension of the axon. We also find a reduction in the amount of filopodia and polymerized actin in *Clasp2* KO growth cones, as compared to control neurons. This is in agreement with a previous report showing that depletion of CLASP in spinal cord neurons of *Xenopus* (which only express one CLASP) leads to a reduction in F-actin in growth cones (Marx et al., 2013). Also in *Xenopus* growth cones, CLASP2 has been reported to modulate the distribution of F-actin structures downstream of Abl (Engel et al., 2014), a tyrosine kinase that binds actin and phosphorylates regulators of the actin cytoskeleton (Colicelli, 2010). CLASP2 also interacts with IQGAP1, a regulator of the actin cytoskeleton (Watanabe et al., 2009). In addition, a number of actin-associated proteins as well as actin-MT crosslinking proteins have been described to be part of the genetic interactome of the *Drosophila* CLASP homolog (Lowery et al., 2010). Thus, in the absence of CLASP2, the disruption of the interactions between CLASP2 and IQGAP1 and other actin regulators, might contribute to actin cytoskeleton defects found in growth cones of *Clasp2* KO neurons.

In summary, we present evidence of opposing functions of CLASP1 and CLASP2 in developing neuronal cells, and we relate them to differential CLASP1 and -2 MT plus-end binding and subcellular localization, as well as their distinct involvement in signaling cascades, both as substrates and upstream regulators. Focusing on CLASP2, we shed light into its role as a negative regulator of neurite and axon extension.

## Author Contributions

CS performed the majority of the experiments, interpreted results, and wrote the manuscript. SB, MR, EB-M, ML, MS, and WI performed experiments and interpreted results. JA interpreted results. NG performed some of the experiments, interpreted results, and wrote the manuscript. All authors helped to complete the manuscript.

## Conflict of Interest Statement

The authors declare that the research was conducted in the absence of any commercial or financial relationships that could be construed as a potential conflict of interest.

## References

[B1] Aher A. Kok M. Sharma A. Rai A. Olieric N. Rodriguez-Garcia R.. (2018). CLASP suppresses microtubule catastrophes through a single TOG domain. Dev. Cell 46, 40.e8–58.e8. 10.1016/j.devcel.2018.05.032 29937387 PMC6035287

[B3] Akhmanova A. Hoogenraad C. C. Drabek K. Stepanova T. Dortland B. Verkerk T.. (2001). Clasps are CLIP-115 and -170 associating proteins involved in the regional regulation of microtubule dynamics in motile fibroblasts. Cell 104, 923–935. 10.1016/s0092-8674(01)00288-4 11290329

[B2] Akhmanova A. Steinmetz M. O. (2015). Control of microtubule organization and dynamics: two ends in the limelight. Nat. Rev. Mol. Cell Biol. 16, 711–726. 10.1038/nrm4084 26562752

[B4] Al-Bassam J. Chang F. (2011). Regulation of microtubule dynamics by TOG-domain proteins XMAP215/Dis1 and CLASP. Trends Cell Biol. 21, 604–614. 10.1016/j.tcb.2011.06.007 21782439 PMC3202638

[B5] Banker G. A. Cowan W. M. (1977). Rat hippocampal neurons in dispersed cell culture. Brain Res. 126, 397–425. 10.1016/0006-8993(77)90594-7 861729

[B6] Beaven R. Dzhindzhev N. S. Qu Y. Hahn I. Dajas-Bailador F. Ohkura H.. (2015). *Drosophila* CLIP-190 and mammalian CLIP-170 display reduced microtubule plus end association in the nervous system. Mol. Biol. Cell 26, 1491–1508. 10.1091/mbc.e14-06-1083 25694447 PMC4395129

[B7] Beffert U. Dillon G. M. Sullivan J. M. Stuart C. E. Gilbert J. P. Kambouris J. A.. (2012). Microtubule plus-end tracking protein CLASP2 regulates neuronal polarity and synaptic function. J. Neurosci. 32, 13906–13916. 10.1523/JNEUROSCI.2108-12.2012 23035100 PMC3489028

[B8] Beurel E. Grieco S. F. Jope R. S. (2015). Glycogen synthase kinase-3 (GSK3): regulation, actions, and diseases. Pharmacol. Ther. 148, 114–131. 10.1016/j.pharmthera.2014.11.016 25435019 PMC4340754

[B9] Bieling P. Kandels-Lewis S. Telley I. A. van Dijk J. Janke C. Surrey T. (2008). CLIP-170 tracks growing microtubule ends by dynamically recognizing composite EB1/tubulin-binding sites. J. Cell Biol. 183, 1223–1233. 10.1083/jcb.200809190 19103809 PMC2606963

[B10] Bieling P. Laan L. Schek H. Munteanu E. L. Sandblad L. Dogterom M.. (2007). Reconstitution of a microtubule plus-end tracking system *in vitro*. Nature 450, 1100–1105. 10.1038/nature06386 18059460

[B11] Cambray-Deakin M. A. Burgoyne R. D. (1987). Acetylated and detyrosinated α-tubulins are co-localized in stable microtubules in rat meningeal fibroblasts. Cell Motil. Cytoskeleton 8, 284–291. 10.1002/cm.970080309 3319198

[B12] Colicelli J. (2010). ABL tyrosine kinases: evolution of function, regulation, and specificity. Sci. Signal. 3:re6. 10.1126/scisignal.3139re6 20841568 PMC2954126

[B13] Dillon G. M. Tyler W. A. Omuro K. C. Kambouris J. Tyminski C. Henry S.. (2017). CLASP2 links reelin to the cytoskeleton during neocortical development. Neuron 93, 1344.e5–1358.e5. 10.1016/j.neuron.2017.02.039 28285824 PMC5405870

[B14] Dotti C. G. Sullivan C. A. Banker G. A. (1988). The establishment of polarity by hippocampal neurons in culture. J. Neurosci. 8, 1454–1468. 10.1523/JNEUROSCI.08-04-01454.1988 3282038 PMC6569279

[B15] Drabek K. Gutiérrez L. Vermeij M. Clapes T. Patel S. R. Boisset J. C.. (2012). The microtubule plus-end tracking protein CLASP2 is required for hematopoiesis and hematopoietic stem cell maintenance. Cell Rep. 2, 781–788. 10.1016/j.celrep.2012.08.040 23084744

[B16] Drabek K. van Ham M. Stepanova T. Draegestein K. van Horssen R. Sayas C. L.. (2006). Role of CLASP2 in microtubule stabilization and the regulation of persistent motility. Curr. Biol. 16, 2259–2264. 10.1016/j.cub.2006.09.065 17113391

[B17] Efimov A. Kharitonov A. Efimova N. Loncarek J. Miller P. M. Andreyeva N.. (2007). Asymmetric CLASP-dependent nucleation of noncentrosomal microtubules at the trans-Golgi network. Dev. Cell 12, 917–930. 10.1016/j.devcel.2007.04.002 17543864 PMC2705290

[B18] Engel U. Zhan Y. Long J. B. Boyle S. N. Ballif B. A. Dorey K.. (2014). Abelson phosphorylation of CLASP2 modulates its association with microtubules and actin. Cytoskeleton 71, 195–209. 10.1002/cm.21164 24520051 PMC4054870

[B19] Etienne-Manneville S. Hall A. (2003). Cdc42 regulates GSK-3β and adenomatous polyposis coli to control cell polarity. Nature 421, 753–756. 10.1038/nature01423 12610628

[B20] Feldman E. L. Sullivan K. A. Kim B. Russell J. W. (1997). Insulin-like growth factors regulate neuronal differentiation and survival. Neurobiol. Dis. 4, 201–214. 10.1006/nbdi.1997.0156 9361296

[B21] Fleming C. E. Mar F. M. Franquinho F. Saraiva M. J. Sousa M. M. (2009). Transthyretin internalization by sensory neurons is megalin mediated and necessary for its neuritogenic activity. J. Neurosci. 29, 3220–3232. 10.1523/JNEUROSCI.6012-08.2009 19279259 PMC6666452

[B22] Honnappa S. Gouveia S. M. Weisbrich A. Damberger F. F. Bhavesh N. S. Jawhari H.. (2009). An EB1-binding motif acts as a microtubule tip localization signal. Cell 138, 366–376. 10.1016/j.cell.2009.04.065 19632184

[B23] Hur E. M. Saijilafu Lee B. D. Kim S. J. Xu W. L. Zhou F. Q. (2011). GSK3 controls axon growth via CLASP-mediated regulation of growth cone microtubules. Genes Dev. 25, 1968–1981. 10.1101/gad.17015911 21937714 PMC3185968

[B24] Kim W. Y. Zhou F. Q. Zhou J. Yokota Y. Wang Y. M. Yoshimura T.. (2006). Essential roles for GSK-3s and GSK-3-primed substrates in neurotrophin-induced and hippocampal axon growth. Neuron 52, 981–996. 10.1016/j.neuron.2006.10.031 17178402 PMC4167845

[B25] Kumar P. Chimenti M. S. Pemble H. Schönichen A. Thompson O. Jacobson M. P.. (2012). Multisite phosphorylation disrupts arginine-glutamate salt bridge networks required for binding of cytoplasmic linker-associated protein 2 (CLASP2) to end-binding protein 1 (EB1). J. Biol. Chem. 287, 17050–17064. 10.1074/jbc.M111.316661 22467876 PMC3366819

[B26] Kumar P. Lyle K. S. Gierke S. Matov A. Danuser G. Wittmann T. (2009). GSK3β phosphorylation modulates CLASP-microtubule association and lamella microtubule attachment. J. Cell Biol. 184, 895–908. 10.1083/jcb.200901042 19289791 PMC2699158

[B27] Langlais P. Dillon J. L. Mengos A. Baluch D. P. Ardebili R. Miranda D. N.. (2012). Identification of a role for CLASP2 in insulin action. J. Biol. Chem. 287, 39245–39253. 10.1074/jbc.m112.394148 22992739 PMC3493964

[B28] Lansbergen G. Grigoriev I. Mimori-Kiyosue Y. Ohtsuka T. Higa S. Kitajima I.. (2006). CLASPs attach microtubule plus ends to the cell cortex through a complex with LL5β. Dev. Cell 11, 21–32. 10.1016/j.devcel.2006.05.012 16824950

[B29] Lanza D. C. Meirelles G. V. Alborghetti M. R. Abrile C. H. Lenz G. Kobarg J. (2010). FEZ1 interacts with CLASP2 and NEK1 through coiled-coil regions and their cellular colocalization suggests centrosomal functions and regulation by PKC. Mol. Cell. Biochem. 338, 35–45. 10.1007/s11010-009-0317-9 19924516

[B30] Laurino L. Wang X. X. de la Houssaye B. A. Sosa L. Dupraz S. Cáceres A.. (2005). PI3K activation by IGF-1 is essential for the regulation of membrane expansion at the nerve growth cone. J. Cell Sci. 118, 3653–3662. 10.1242/jcs.02490 16046480

[B31] Lawrence E. J. Arpag G. Norris S. R. Zanic M. (2018). Human CLASP2 specifically regulates microtubule catastrophe and rescue. Mol. Biol. Cell 29, 1168–1177. 10.1091/mbc.E18-01-0016 29540526 PMC5935067

[B32] Lee H. Engel U. Rusch J. Scherrer S. Sheard K. Van Vactor D. (2004). The microtubule plus end tracking protein Orbit/MAST/CLASP acts downstream of the tyrosine kinase Abl in mediating axon guidance. Neuron 42, 913–926. 10.1016/j.neuron.2004.05.020 15207236

[B33] Lowery L. A. Lee H. Lu C. Murphy R. Obar R. A. Zhai B.. (2010). Parallel genetic and proteomic screens identify Msps as a CLASP-Abl pathway interactor in *Drosophila*. Genetics 185, 1311–1325. 10.1534/genetics.110.115626 20498300 PMC2927758

[B34] Marx A. Godinez W. J. Tsimashchuk V. Bankhead P. Rohr K. Engel U. (2013). *Xenopus* cytoplasmic linker-associated protein 1 (XCLASP1) promotes axon elongation and advance of pioneer microtubules. Mol. Biol. Cell 24, 1544–1558. 10.1091/mbc.E12-08-0573 23515224 PMC3655815

[B35] Matsui T. Watanabe T. Matsuzawa K. Kakeno M. Okumura N. Sugiyama I.. (2015). PAR3 and aPKC regulate Golgi organization through CLASP2 phosphorylation to generate cell polarity. Mol. Biol. Cell 26, 751–761. 10.1091/mbc.E14-09-1382 25518939 PMC4325844

[B36] Mimori-Kiyosue Y. Grigoriev I. Lansbergen G. Sasaki H. Matsui C. Severin F.. (2005). CLASP1 and CLASP2 bind to EB1 and regulate microtubule plus-end dynamics at the cell cortex. J. Cell Biol. 168, 141–153. 10.1083/jcb.200405094 15631994 PMC2171674

[B37] Neukirchen D. Bradke F. (2011). Cytoplasmic linker proteins regulate neuronal polarization through microtubule and growth cone dynamics. J. Neurosci. 31, 1528–1538. 10.1523/JNEUROSCI.3983-10.2011 21273437 PMC6623617

[B38] Pfenninger K. H. Laurino L. Peretti D. Wang X. Rosso S. Morfini G.. (2003). Regulation of membrane expansion at the nerve growth cone. J. Cell Sci. 116, 1209–1217. 10.1242/jcs.00285 12615964

[B39] Ruiz-Saenz A. van Haren J. Sayas C. L. Rangel L. Demmers J. Millán J.. (2013). Protein 4.1R binds to CLASP2 and regulates dynamics, organization and attachment of microtubules to the cell cortex. J. Cell Sci. 126, 4589–4601. 10.1242/jcs.120840 23943871

[B40] Schneider C. A. Rasband W. S. Eliceiri K. W. (2012). NIH Image to ImageJ: 25 years of image analysis. Nat. Methods 9, 671–675. 10.1038/nmeth.2089 22930834 PMC5554542

[B41] Shi S. H. Cheng T. Jan L. Y. Jan Y. N. (2004). APC and GSK-3β are involved in mPar3 targeting to the nascent axon and establishment of neuronal polarity. Curr. Biol. 14, 2025–2032. 10.1016/j.cub.2004.11.009 15556865

[B42] Shi S. H. Jan L. Y. Jan Y. N. (2003). Hippocampal neuronal polarity specified by spatially localized mPar3/mPar6 and PI 3-kinase activity. Cell 112, 63–75. 10.1016/s0092-8674(02)01249-7 12526794

[B43] Stepanova T. Slemmer J. Hoogenraad C. C. Lansbergen G. Dortland B. De Zeeuw C. I.. (2003). Visualization of microtubule growth in cultured neurons via the use of EB3-GFP (end-binding protein 3-green fluorescent protein). J. Neurosci. 23, 2655–2664. 10.1523/JNEUROSCI.23-07-02655.2003 12684451 PMC6742099

[B44] Trapnell C. Pachter L. Salzberg S. L. (2009). TopHat: discovering splice junctions with RNA-Seq. Bioinformatics 25, 1105–1111. 10.1093/bioinformatics/btp120 19289445 PMC2672628

[B45] Trapnell C. Williams B. A. Pertea G. Mortazavi A. Kwan G. van Baren M. J.. (2010). Transcript assembly and quantification by RNA-Seq reveals unannotated transcripts and isoform switching during cell differentiation. Nat. Biotechnol. 28, 511–515. 10.1038/nbt.1621 20436464 PMC3146043

[B46] van Rossum A. G. de Graaf J. H. Schuuring-Scholtes E. Kluin P. M. Fan Y. X. Zhan X.. (2003). Alternative splicing of the actin binding domain of human cortactin affects cell migration. J. Biol. Chem. 278, 45672–45679. 10.1074/jbc.M306688200 12952985

[B47] van Weering D. H. de Rooij J. Marte B. Downward J. Bos J. L. Burgering B. M. (1998). Protein kinase B activation and lamellipodium formation are independent phosphoinositide 3-kinase-mediated events differentially regulated by endogenous Ras. Mol. Cell. Biol. 18, 1802–1811. 10.1128/mcb.18.4.1802 9528752 PMC121410

[B48] Watanabe T. Noritake J. Kakeno M. Matsui T. Harada T. Wang S.. (2009). Phosphorylation of CLASP2 by GSK-3β regulates its interaction with IQGAP1, EB1 and microtubules. J. Cell Sci. 122, 2969–2979. 10.1242/jcs.046649 19638411

[B49] Welsh G. I. Proud C. G. (1993). Glycogen synthase kinase-3 is rapidly inactivated in response to insulin and phosphorylates eukaryotic initiation factor eIF-2B. Biochem. J. 294, 625–629. 10.1042/bj2940625 8397507 PMC1134506

[B50] Witte H. Neukirchen D. Bradke F. (2008). Microtubule stabilization specifies initial neuronal polarization. J. Cell Biol. 180, 619–632. 10.1083/jcb.200707042 18268107 PMC2234250

[B51] Wittmann T. Waterman-Storer C. M. (2005). Spatial regulation of CLASP affinity for microtubules by Rac1 and GSK3β in migrating epithelial cells. J. Cell Biol. 169, 929–939. 10.1083/jcb.200412114 15955847 PMC2171640

[B52] Wu X. Shen Q. T. Oristian D. S. Lu C. P. Zheng Q. Wang H. W.. (2011). Skin stem cells orchestrate directional migration by regulating microtubule-ACF7 connections through GSK3β. Cell 144, 341–352. 10.1016/j.cell.2010.12.033 21295697 PMC3050560

[B53] Yoshimura T. Kawano Y. Arimura N. Kawabata S. Kikuchi A. Kaibuchi K. (2005). GSK-3β regulates phosphorylation of CRMP-2 and neuronal polarity. Cell 120, 137–149. 10.1016/j.cell.2004.11.012 15652488

[B54] Yu N. Signorile L. Basu S. Ottema S. Lebbink J. H. G. Leslie K.. (2016). Isolation of functional tubulin dimers and of tubulin-associated proteins from mammalian cells. Curr. Biol. 26, 1728–1736. 10.1016/j.cub.2016.04.069 27291054

[B55] Zhou F. Q. Zhou J. Dedhar S. Wu Y. H. Snider W. D. (2004). NGF-induced axon growth is mediated by localized inactivation of GSK-3β and functions of the microtubule plus end binding protein APC. Neuron 42, 897–912. 10.1016/j.neuron.2004.05.011 15207235

