# Supplementary material for: Distinct Functions for Mammalian CLASP1 and -2 During Neurite and Axon Elongation
Source: Front Cell Neurosci. 2019 Jan 29;13:5. doi: 10.3389/fncel.2019.00005 (PMC6373834; doi:10.3389/fncel.2019.00005)
Supplement: Supplementary file 1 [file Image_1.pdf]

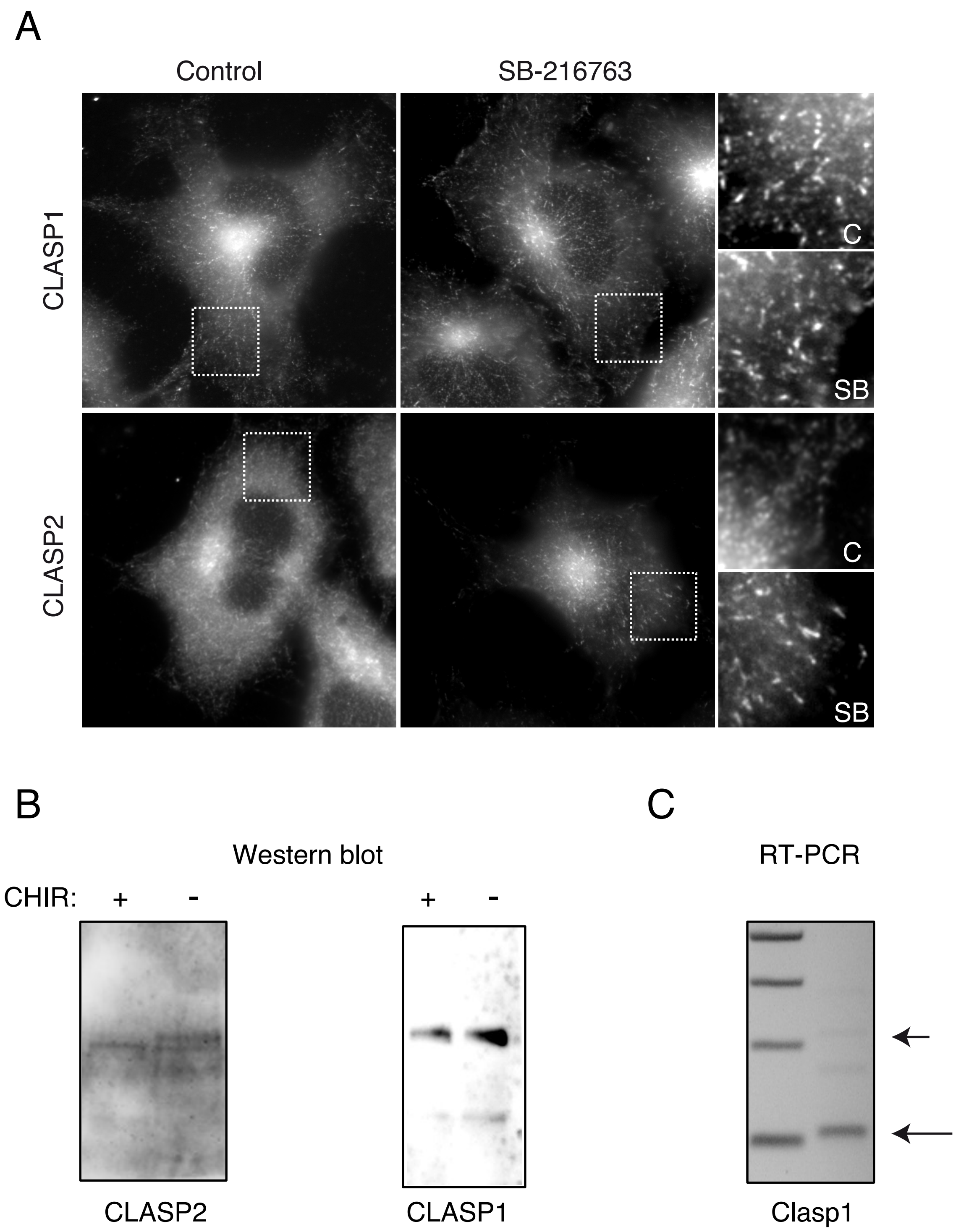

Figure S1. Different responses of CLASP1 and -2 to GSK3.
A) N1E-115 cells were serum starved overnight and treated with vehicle (Control) or SB-216763 (25 uM, 30 min.), fixed and stained with specific antibodies against CLASP1 or CLASP2. SB-216763, a specific GSK-3 inhibitor, dramatically enhances CLASP2 accumulation at MT plus-ends, whereas it reduces the amount of CLASP1-highlighted MT-comets. **B**) N1E-115 cells were serum-starved overnight and subsequently either treated for 3 hours with the GSK3 inhibitor CHIR (+), or not treated (-). Cell lysates were examined for the presence of posttranslationally modified forms of CLASP1 and -2 using Western blots and specific anti-CLASP1/2 antibodies. In control serum-starved cells we detected two CLASP2 bands, which we assume to represent phosphorylated and non-phosphorylated forms of CLASP2. In cells treated with CHIR only the lower band was detected. We only detected one CLASP1 protein, irrespective of whether cells were treated with CHIR or not. **C**) RT-PCR on mRNA derived from N1E-115 cells reveals alternative splicing of a small exon in the *Clasp1* gene. PCR was performed with primers surrounding the exon. The large arrow points to an amplified fragment lacking the exon, the small arrow points to the amplified fragment containing the exon.
