# Supplementary material for: Distinct Functions for Mammalian CLASP1 and -2 During Neurite and Axon Elongation
Source: Front Cell Neurosci. 2019 Jan 29;13:5. doi: 10.3389/fncel.2019.00005 (PMC6373834; doi:10.3389/fncel.2019.00005)
Supplement: Supplementary file 2 [file Image_2.pdf]

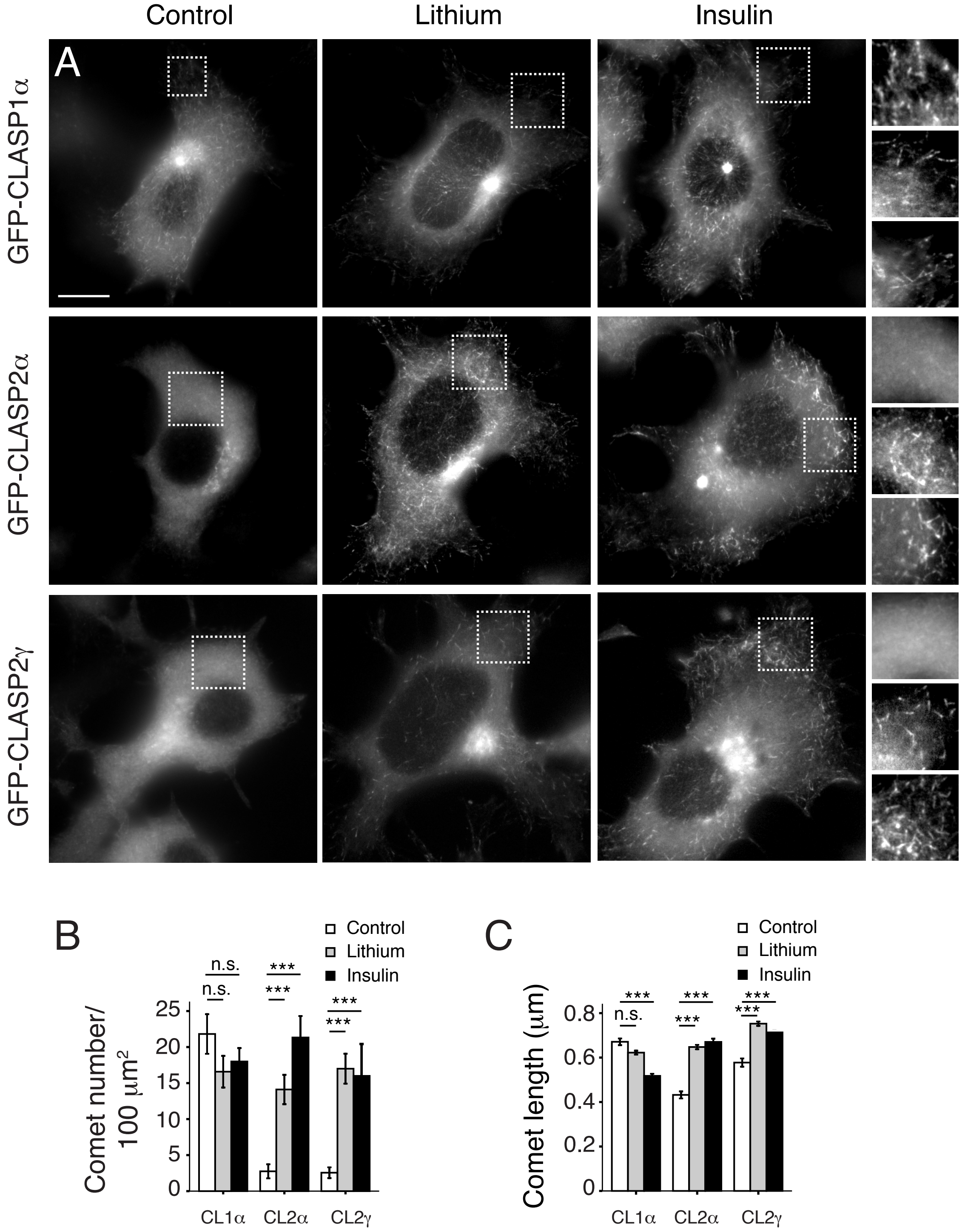

Figure S2. Differential regulation of GFP-CLASPs by GSK3 and Insulin.

A) N1E-115 cells were transfected with GFP-CLASP1α (upper panels), GFP-CLASP2α (middle panels) or GFP-CLASP2γ (lower panels), and treated with lithium chloride (20 mM) and myo-Inositol (10 mM) for 4 hours (Lithium), or insulin (2 μg/ml) for 10 min (before the treatments, cells were serum starved overnight). Cells were fixed after treatments. Fluorescence images show control and treated cells expressing low levels of GFP-tagged CLASPs. Scale bar= 20 μm. **B**) Quantification of the number of GFP-CLASP comets/100 μm^2. **C**) Quantification of the length of GFP-CLASP-comets (in μm). SEM is depicted in the bar graphs. Anova tests were performed in B) and C).
