# Supplementary material for: Distinct Functions for Mammalian CLASP1 and -2 During Neurite and Axon Elongation
Source: Front Cell Neurosci. 2019 Jan 29;13:5. doi: 10.3389/fncel.2019.00005 (PMC6373834; doi:10.3389/fncel.2019.00005)
Supplement: Supplementary file 3 [file Image_3.pdf]

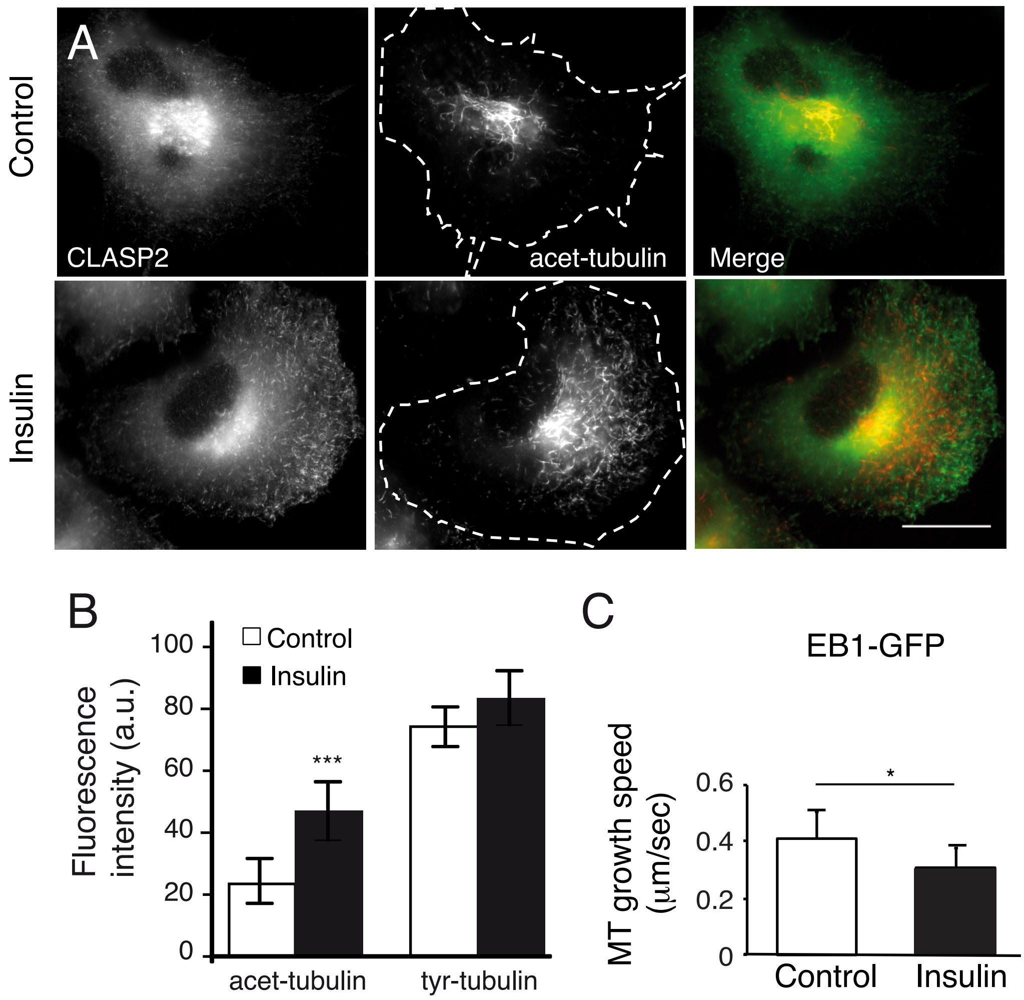

Figure S3. Insulin increases the level of acetylated (stable) MTs.
A) Insulin leads to CLASP2 relocalization to MT plus-ends at cell edges in correlation with an increase in acetylated tubulin. Control and insulin-stimulated cells, treated as described above, were fixed and double stained with anti-CLASP2 (green in merge) and anti-acetylated tubulin (red in merge) antibodies. **B**) Quantification of average fluorescence intensities (± SD) of acetylated- and tyrosinated-tubulin in control versus insulin-treated cells is shown. Scale bars = 20 μm. **C**) Quantification of MT growth rates in Control and Insulin-treated cells, as measured with EB1-GFP. MT growth speed is reduced upon Insulin treatment. Average growth rates ± SD are depicted. Student T test was performed.
