# Supplementary material for: Distinct Functions for Mammalian CLASP1 and -2 During Neurite and Axon Elongation
Source: Front Cell Neurosci. 2019 Jan 29;13:5. doi: 10.3389/fncel.2019.00005 (PMC6373834; doi:10.3389/fncel.2019.00005)
Supplement: Supplementary file 4 [file Image_4.pdf]

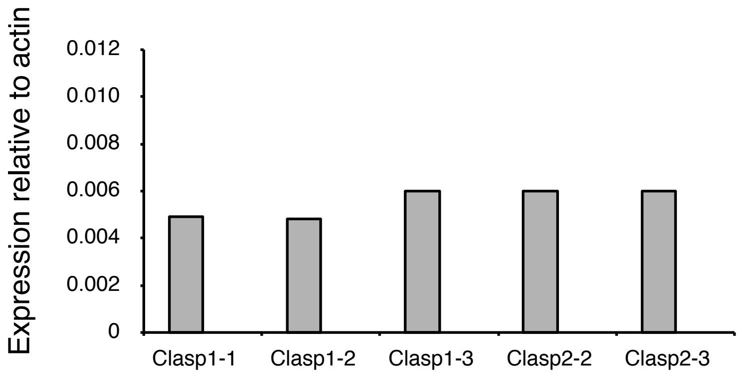

Figure S4. Expression of *Clasp1* and *-2* in N1E-115 cells.
RT-PCR analysis comparing *Clasp1* and *Clasp2* mRNA levels in N1E-115 cells. Three primer sets were used for *Clasp1*, two sets were used for *Clasp2*.
