# Supplementary material for: Distinct Functions for Mammalian CLASP1 and -2 During Neurite and Axon Elongation
Source: Front Cell Neurosci. 2019 Jan 29;13:5. doi: 10.3389/fncel.2019.00005 (PMC6373834; doi:10.3389/fncel.2019.00005)
Supplement: Supplementary file 5 [file Image_5.pdf]

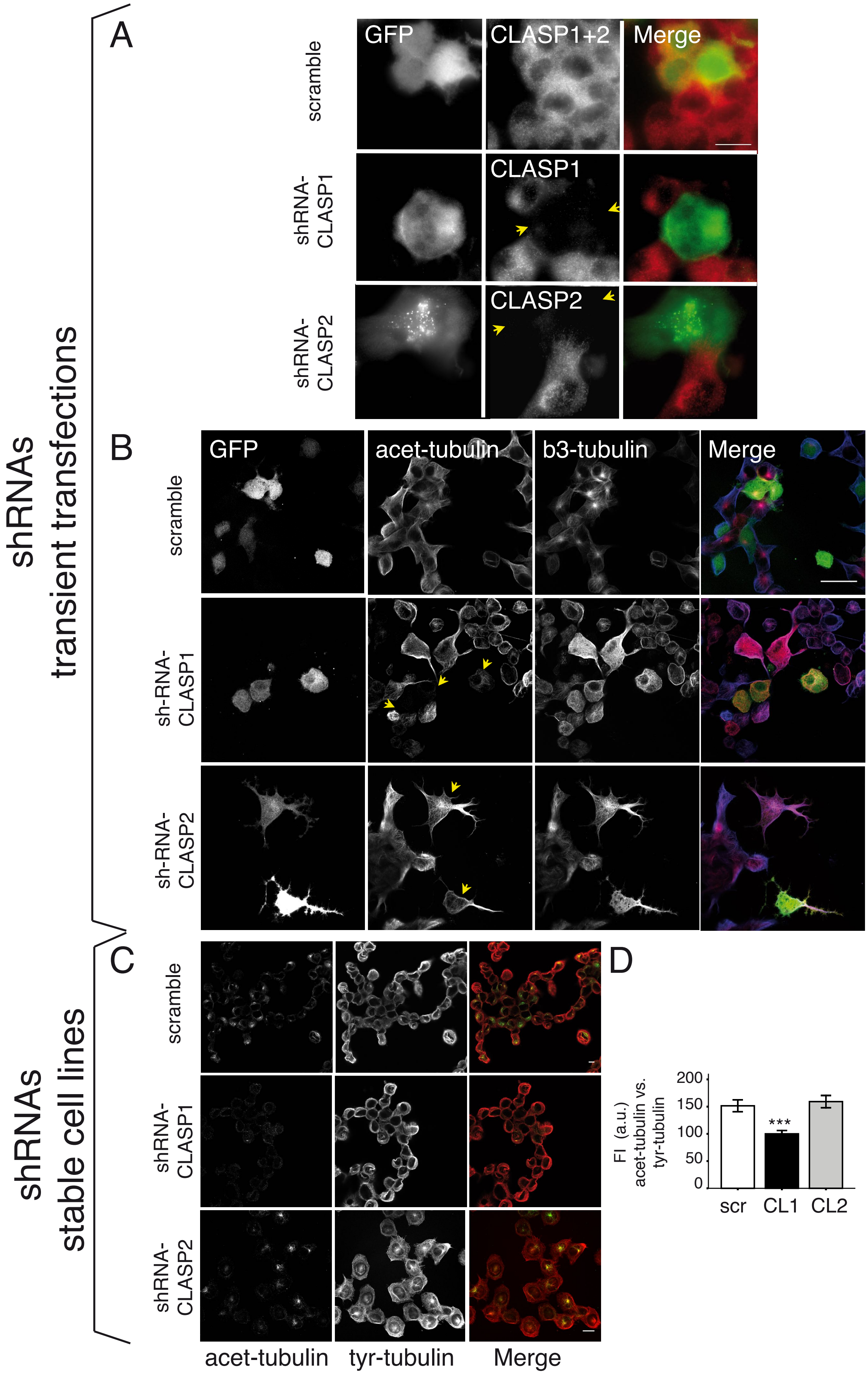

shRNAs
transient transfections
shRNAs
stable cell lines
A
GFP
CLASP1+2
Merge
scramble
shRNA-CLASP1
CLASP1
shRNA-CLASP2
CLASP2
B
GFP
acet-tubulin
b3-tubulin
Merge
scramble
sh-RNA-CLASP1
sh-RNA-CLASP2
C
scramble
shRNA-CLASP1
shRNA-CLASP2
acet-tubulin
tyr-tubulin
Merge
D
FI (a.u.) acet-tubulin vs. tyr-tubulin
200
150
100
50
0
***
scr
CL1
CL2

Figure S5. Knockdown of CLASP1 and CLASP2 in N1E-115 cells.
**A, B**) N1E-115 cells were transiently transfected with GFP-bearing shRNA constructs against either CLASP1 or CLASP2. A scramble (non-targeting) shRNA was used as a control (upper panel). Cells were fixed 72 hours post-transfection (non-subject to serum starvation). **A**) Fluorescence images showing N1E-115 cells, stained with antibodies against CLASP1 and CLASP2 (Control, upper panel), CLASP1 (CLASP1-depleted, middle panel), or CLASP2 (CLASP2-depleted, lower panel). These stainings confirm that, while control green cells show normal levels of CLASPs, green cells in which shRNAs against CLASPs were used show either CLASP1 or CLASP2 downregulation. Scale bar = 20 μm. **B**) CLASP1-depleted cells show rounded morphology and a reduction in acetylated tubulin levels, whereas CLASP2-depleted cells present a differentiated morphology (flattened cells and cells with neurites), with no differences in the levels of acetylated MTs. Confocal fluorescence images show cells stained with anti-acetylated-tubulin (red in merge) and anti-BIII-tubulin (blue in merge). GFP-transfected cells are visualized in green. Scale bar = 50 μm. **C, D**) Stable depletion of CLASP1 or CLASP2 leads to different effects on cell morphology and MT stability. N1E-115 cells were subjected to lentiviral transduction with scramble shRNA (Control) or shRNAs targeting either CLASP1 or CLASP2. Stable cell lines deficient in each CLASP were generated by puromycin selection for two weeks. In (D), confocal images of Control cells (upper panel) or cells stably depleted in either CLASP1 (middle panel) or CLASP2 (lower panel), stained with antibodies against acetylated-tubulin (green in merge, stable MTs), or tyrosinated-tubulin (red in merge, dynamic MTs). Scale bar= 20 μm. **D**) Quantification of the fluorescence intensity (in a.u.) of the levels of acetylated versus tyrosinated tubulin in Control (scramble), CLASP1- and CLASP2-stably depleted cells. SD is shown in bar graphs.
