# Supplementary material for: Distinct Functions for Mammalian CLASP1 and -2 During Neurite and Axon Elongation
Source: Front Cell Neurosci. 2019 Jan 29;13:5. doi: 10.3389/fncel.2019.00005 (PMC6373834; doi:10.3389/fncel.2019.00005)
Supplement: Supplementary file 6 [file Image_6.pdf]

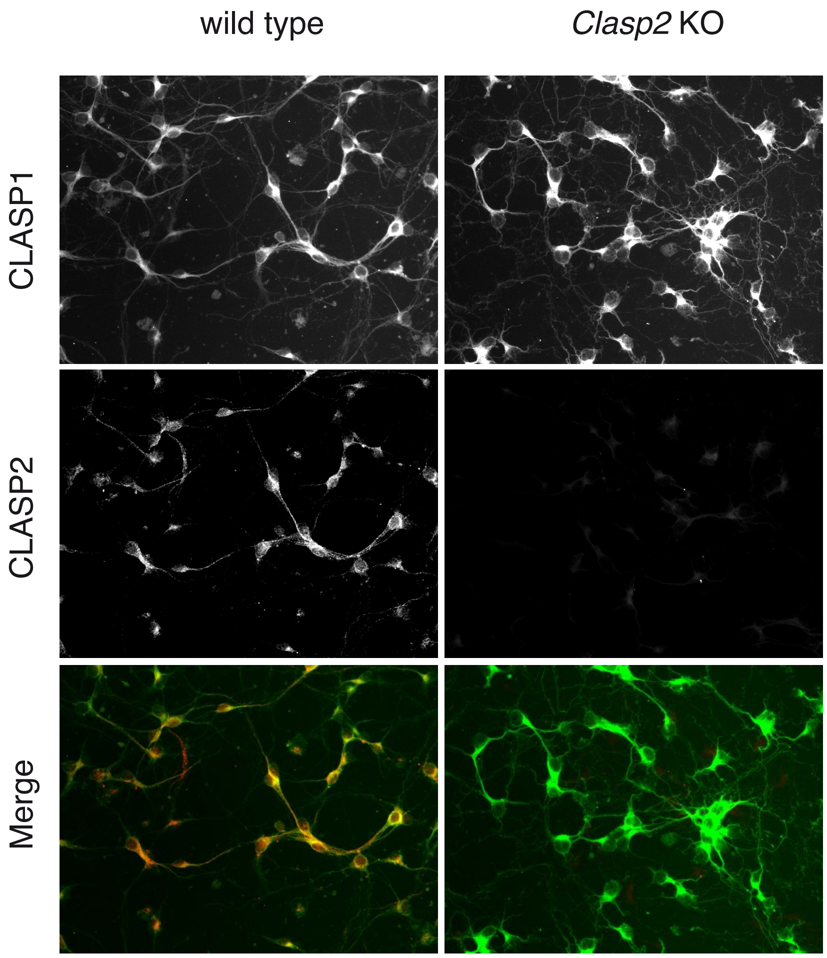

Figure S6. CLASP2 depletion in *Clasp2* knockout neurons.
Fluorescence images of primary hippocampal 1DIV wild type and *Clasp2* knockout (KO) neurons, stained with anti-CLASP1 and anti-CLASP2 antisera.
