# Supplementary material for: Distinct Functions for Mammalian CLASP1 and -2 During Neurite and Axon Elongation
Source: Front Cell Neurosci. 2019 Jan 29;13:5. doi: 10.3389/fncel.2019.00005 (PMC6373834; doi:10.3389/fncel.2019.00005)
Supplement: Supplementary file 7 [file Image_7.pdf]

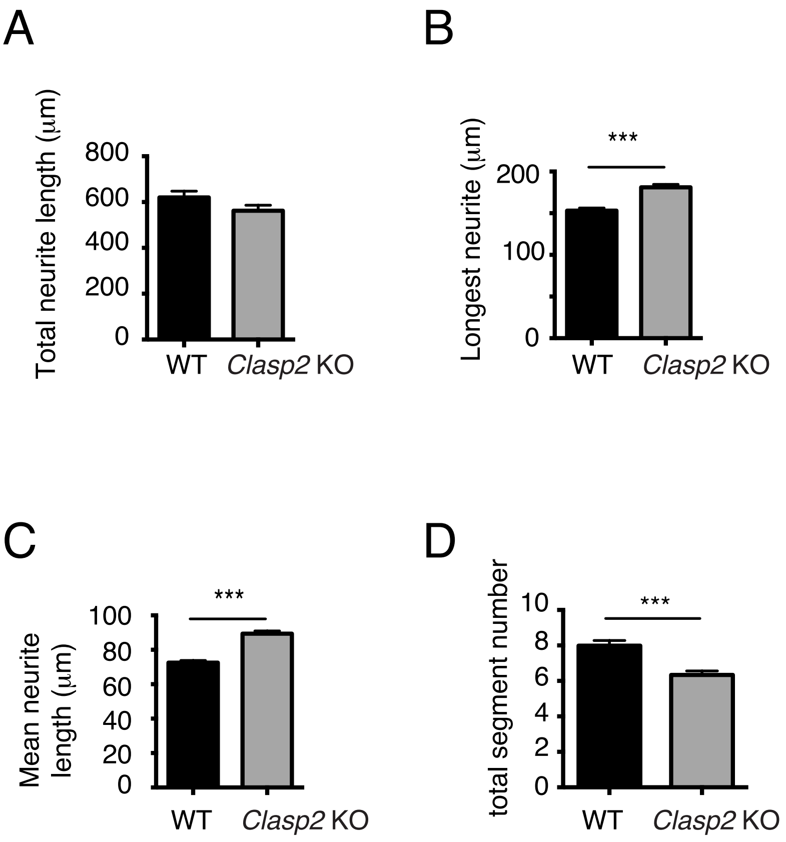

Figure S7. *Clasp2* KO induces an increase in neurite length in DRG neurons.
Quantification of total neurite length (μm) (**A**), longest neurite (μm) (**B**), mean neurite length (μm) (**C**) and number of segments (**D**) in primary cultures of dorsal root ganglia (DRG) neurons. To measure neurite outgrowth, IF for βIII-tubulin was performed.
