# Supplementary material for: Distinct Functions for Mammalian CLASP1 and -2 During Neurite and Axon Elongation
Source: Front Cell Neurosci. 2019 Jan 29;13:5. doi: 10.3389/fncel.2019.00005 (PMC6373834; doi:10.3389/fncel.2019.00005)
Supplement: Supplementary file 8 [file Image_8.pdf]

A

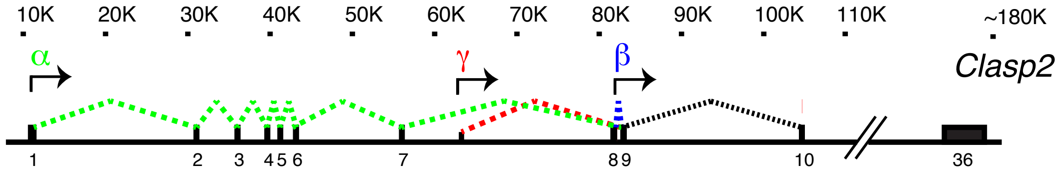

B

	α	γ	β
% reads in HN	0	27	73
% reads in ES	100	0	0

C

gene	mRNA HN	mRNA DRG
Clasp1	18	21
Clasp2	51	65
Mapre1	78	30
Clip1	10	10

Figure S8. Analysis of *Clasp1* and -*2* expression in neurons.
A) Schematic representation of the *Clasp2* gene in mouse. Alternative promoter usage gives rise to *Clasp2*α (green), -β (blue), and -γ (red) mRNAs. **B, C**) Quantification of reads based on RNA-Seq experiments using RNA derived from hippocampal neurons (HN), embryonic stem cells (ES), or DRG neurons. In C, the genes encoding EB1 (*Mapre1*) and CLIP-170 (*Clip1*) are shown for comparison.
