# Supplementary material for: Distinct Functions for Mammalian CLASP1 and -2 During Neurite and Axon Elongation
Source: Front Cell Neurosci. 2019 Jan 29;13:5. doi: 10.3389/fncel.2019.00005 (PMC6373834; doi:10.3389/fncel.2019.00005)
Supplement: Supplementary file 9 [file Image_9.pdf]

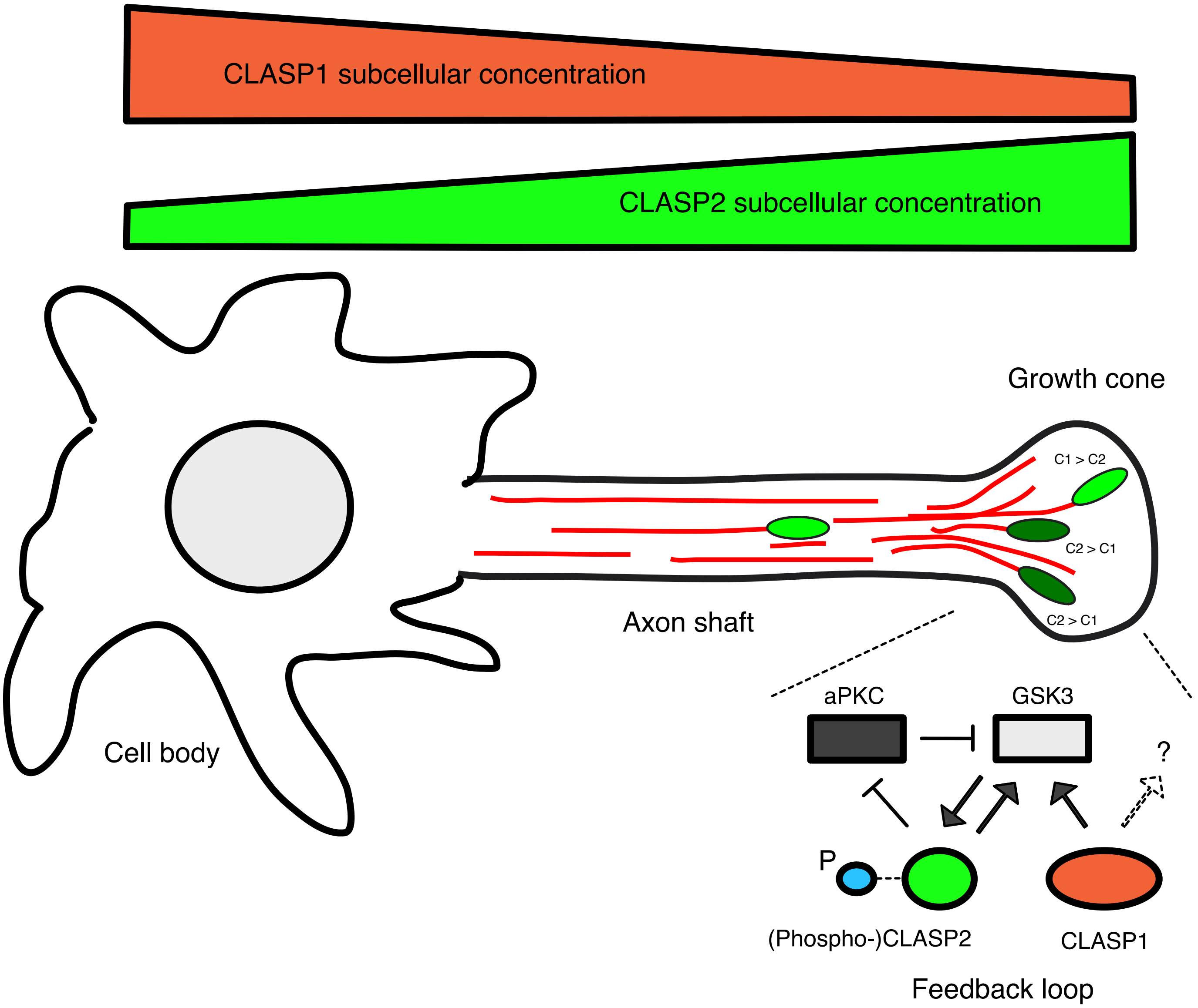

Figure S9. Model of CLASP1 and -2 function during axon extension.
Schematic representation of a neuron with a growing axon and extending growth cone. Microtubules (MTs, red lines) are present throughout the axon and growth cone, with the vast majority of MT plus ends pointing in the direction of the growth cone (note that MTs have not been drawn in the cell body). The growth cone has more dynamic MTs than the axon. The ends of growing MTs are decorated with +TIPs (green ellipses), including the CLASPs. Our data suggest that although CLASP1 and -2 are present throughout the neuron, CLASP1 is more concentrated in the cell body and the proximal region of the axon, whereas CLASP2 is more enriched in the growth cone. The major CLASP isoforms in the growth cone are CLASP1a (C1) and CLASP2g (C2). We propose that these have different functions, i.e. CLASP1a mainly promotes MT growth, whereas CLASP2g mainly promotes MT stabilization by attaching growing MTs to proteins at the cell cortex. The latter activity is regulated by GSK3, which phosphorylates CLASP2 and causes its release from MT ends. We hypothesize that on a persistently growing MT that invades the growth cone periphery, or at proximal regions of the neurite/axon shaft, where GSK3 is largely active, the amount of CLASP1a on the MT end is higher (C1 > C2). By contrast, on a stabilized MT that grows slower (indicated by the dark green ellipses), and where GSK3 might be largely inactive, the amount of non-phosphorylated CLASP2g at MT ends is higher (C2 > C1). CLASPs are furthermore involved in complex feedback signalling loops, i.e. depletion of CLASP2 activates aPKC, which is upstream of GSK3, and inactivates this kinase, and depletion of either CLASP1 or -2 causes phosphorylation (and hence inactivation) of GSK3. CLASP1 may also act on upstream factors, however, we have not addressed this in our study (stippled arrow and question mark). Thus, GSK3 phosphorylation and inactivation appears to be both downstream of signaling cascades triggered at growth factor receptors and downstream of CLASP themselves. In addition, phosphorylated CLASP2 could compete with non-phosphorylated CLASP2 for cortical binding sites.
